# Efficacy and Safety of Dual Antiplatelet Therapy in Patients Undergoing Coronary Stent Implantation: A Systematic Review and Network Meta-Analysis

**DOI:** 10.1155/2021/9934535

**Published:** 2021-05-05

**Authors:** Yi Xu, Yimin Shen, Delong Chen, Pengfei Zhao, Jun Jiang

**Affiliations:** ^1^Department of Cardiology, The Second Affiliated Hospital of Zhejiang University School of Medicine, 88 Jiefang Road, Hangzhou, Zhejiang 310009, China; ^2^School of Chinese Materia Medica, Nanjing University of Chinese Medicine, 138 Xianlin Avenue, Nanjing 210023, China; ^3^State Key Laboratory of Drug Research, Shanghai Institute of Materia Medica, Chinese Academy of Sciences, 501 Haike Road, Shanghai 201203, China

## Abstract

**Introduction:**

This network meta-analysis aimed to evaluate the efficacy and safety of different dual antiplatelet therapies (DAPTs) after percutaneous coronary intervention (PCI) with drug-eluting stents (DESs).

**Methods:**

Randomized controlled trials (RCTs) comparing longer-term (>12 months) DAPT (L-DAPT), 12-month DAPT (DAPT 12Mo), 6-month DAPT (DAPT 6Mo), 3-month DAPT followed by aspirin monotherapy (DAPT 3Mo + ASA), 3-month DAPT followed by a P2Y12 receptor inhibitor monotherapy (DAPT 3Mo + P2Y12), or 1-month DAPT with a P2Y12 receptor inhibitor monotherapy (DAPT 1Mo + P2Y12) were searched. Primary endpoints were all-cause mortality, cardiac death, myocardial infarction (MI), major bleeding, any bleeding, definite or probable stent thrombosis (ST), and net adverse clinical events (NACE). This Bayesian network meta-analysis was performed with the random-effects model.

**Results:**

Twenty-four RCTs (*n* = 81339) were included. In comparison with L-DAPT, DAPT 6Mo (OR: 0.50, 95% CI: 0.29–0.83), DAPT 3Mo + P2Y12 (OR: 0.38, 95% CI: 0.18–0.82), DAPT 3Mo + ASA (OR: 0.44, 95% CI: 0.17–0.98), and DAPT 1Mo + P2Y12 (OR: 0.45, 95% CI: 0.14–0.93) were associated with a lower risk of major bleeding. DAPT 3Mo + P2Y12 (OR: 0.58, 95% CI: 0.38–0.88) reduced the risk of any bleeding when compared with DAPT 12Mo. L-DAPT decreased the risk of MI and definite or probable stent ST when compared with DAPT 6Mo. DAPT 3Mo + P2Y12 decreased the risk of NACE in comparison with DAPT 6Mo and DAPT 12Mo. No significant difference in all-cause mortality and cardiac death was observed. In patients with acute coronary syndrome, DAPT 6Mo was comparable to DAPT 12Mo.

**Conclusion:**

Short-term (1–3 months) DAPT is noninferior to DAPT 6Mo after DESs implantation, while L-DAPT reduces MI and definite or probable ST rates. DAPT 3Mo + P2Y12 might be a reasonable trade-off in patients with high risk of bleeding accompanied by ischemia.

## 1. Introduction

Dual antiplatelet therapy (DAPT) consisting of aspirin and a P2Y12 receptor inhibitor is the foundation of secondary prevention for patients with coronary artery disease (CAD) after drug-eluting stents (DESs) implantation [1, 2]. Nevertheless, the optimal duration of DAPT continues to be controversial.

In recent decades, the development of stent technologies and the popularization of more potent P2Y12 receptor inhibitors have witnessed a decline in the incidence of ischemic complications [3, 4]. In patients with stable CAD, the 2016 ACC/AHA and the 2017 ESC guidelines both recommended 6-month DAPT with the indefinite continuation of aspirin monotherapy after DES implantation [1, 2]. Furthermore, a prolonged DAPT course >12 months should be considered in patients with high thrombotic risk, and discontinuation of DAPT at 3 months might be an alternative in those with high bleeding risk [1, 2].

Five recent RCTs explored the benefit of ≤3-month DAPT with a P2Y12 receptor inhibitor monotherapy [5–9]. Among them, the TICO trial enrolled patients with acute coronary syndrome (ACS), and the TWILIGHT trial enrolled patients with high bleeding or ischemic risk [5, 7]. 2020 ESC guidelines considered discontinuation of aspirin after 3–6 months in patients with non-ST-segment elevation ACS, depending on the balance between bleeding and ischemia [10]. However, owing to the limitation of finite randomized comparisons, the efficacy and safety of 1 or 3 months DAPT compared with other strategies remain uncertain in acute or chronic coronary syndrome.

Thus, we performed a network meta-analysis to evaluate different regimens of DAPT in patients undergoing PCI with DESs.

## 2. Methods

This network meta-analysis was performed in accordance with the Preferred Reporting Items for Systematic Reviews and Meta-Analysis (PRISMA) guidelines (Supplementary Table S1) [11].

### 2.1. Search Strategy and Selection Criteria

Two investigators (Y.S. and D.C.) systematically and independently searched five databases (PubMed, Embase, the Cochrane Library, Web of Science, and ClinicalTrials.gov) without language restrictions from their inception to 22 September 2020. The following keywords and controlled vocabularies were used: “platelet aggregation inhibitors,” “aspirin,” “drug-eluting stent,” “percutaneous coronary intervention,” and “randomized controlled trial” (Supplementary Table S2). Presentations about unpublished relevant RCTs of important international conferences were verified simultaneously for additional literature to guarantee a comprehensive search.

Eligible RCTs were supposed to meet the following inclusion criteria: (1) participants were adults with CAD who received DAPT after undergoing PCI with DESs implantation; (2) the interventions corresponded with the following candidate therapies: longer-term (>12 months) DAPT (L-DAPT), 12-month DAPT (DAPT 12Mo), 6-month DAPT (DAPT 6Mo), 3-month DAPT followed by aspirin monotherapy (DAPT 3Mo + ASA), 3-month DAPT followed by a P2Y12 receptor inhibitor monotherapy (DAPT 3Mo + P2Y12), or 1-month DAPT with a P2Y12 receptor inhibitor monotherapy (DAPT 1Mo + P2Y12); (3) outcomes of efficacy endpoints were available; (4) studies beyond 12-month follow-up.

We excluded studies that met the following criteria: (1) nonrandomized trials; (2) trials used predominantly bare-metal stents (BMSs); (3) trials with a crossover design.

### 2.2. Data Extraction and Quality Assessment

Two investigators (Y. X. and P. Z.) independently screened the titles, abstracts, and sequentially full articles. Then, they extracted data on the study design, baseline characteristics, and outcomes from full texts or published appendixes using prespecified forms. The efficacy endpoints included all-cause mortality, cardiac death, myocardial infarction (MI), major bleeding, any bleeding, definite or probable stent thrombosis (ST), and net adverse clinical events (NACE). We gave preference to data from long-term or mature follow-up of premier trials. Data extraction was under the instruction of the intention-to-treat principle. We appraised the quality of eligible studies according to the Cochrane Risk of Bias Tool [12]. Moreover, a third investigator (J. J.) identified the accuracy of the information and handled the contradictions by consensus.

### 2.3. Data Synthesis and Statistical Analysis

We applied odds ratios (OR) with corresponding 95% confidence intervals (CI) to demonstrate time-to-event data quantifying the contributions of each duration. 95% CI that did not cross one was considered statistically significant. We pooled evidence within the Bayesian framework for its superiority in reconciling complex conditions by using the Aggregate Data Drug Information System (ADDIS version 1.16.8). The network plots and funnel plots were drawn by Stata version 15.1 using the “networkplot” and “netfunnel” commands.

First, we conducted a pair-wise meta-analysis to aggregate the data of different treatments in terms of the efficacy endpoints with a random-effects model (Supplementary Figure S1). The heterogeneity of direct comparisons was assessed by the *I*^2^ statistic [13]. An *I*^2^ value of smaller than 25% indicated low heterogeneity, 25–50% indicated moderate heterogeneity, and above 50% indicated high heterogeneity. The *p* value of 0.05 was identified as statistical significance. Subsequently, we fitted a Bayesian random-effects network meta-analysis model using Markov chain Monte Carlo (MCMC) algorithms to compare multiple therapies simultaneously [14]. Models were calculated with MCMC simulations, using 4 chains with overdispersed values, with Gibbs sampling based on 50,000 simulation iterations after 20,000 tuning iterations. Convergence was assessed by calculating the Potential Scale Reduction Factor (PSRF, the value < 1.2 was acceptable) according to the Brooks-Gelman-Rubin method. Rank probabilities of each intervention were calculated and were positively related to values. Consistency could be verified when the values of inconsistency factors were close to 0, the values of variance calculation were approximately equal, and the values of inconsistency and consistency models were equivalent [15]. The node-splitting approach was subsequently performed to evaluate consistency [16]. We applied funnel plots to evaluate publication bias. We performed a sensitivity analysis after removing the SMART-DATE trial to confirm the reliability of our outcomes. Besides, prespecified subgroup analyses were performed for patients with ACS and undergoing newer-generation DESs implantation.

## 3. Results

### 3.1. Search Results

**Figure 1:**
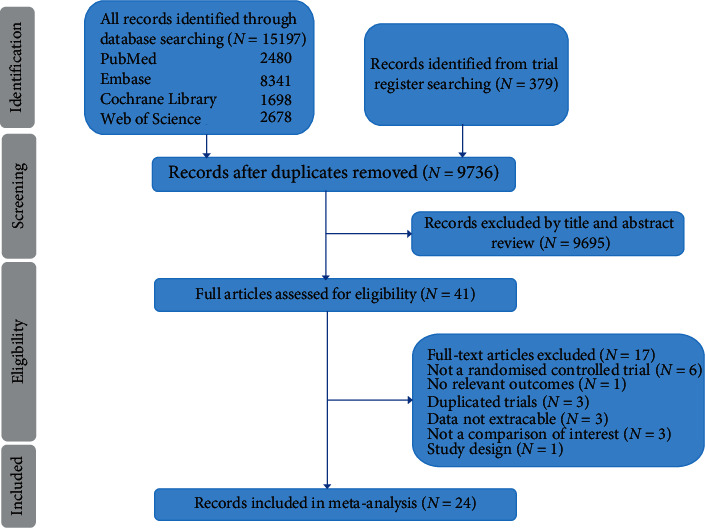
PRISMA flow diagram detailing study selection.

The systematic search outputted 15197 articles, and 9736 of them were subsequently screened after removing duplicates. Then, 41 trials were appraised as full text. Eventually, 24 randomized controlled trials with altogether 81339 participants were deemed eligible for inclusion criteria (Figure 1) [5–9, 17–35].

### 3.2. Characteristics of Included Studies and Bias Assessment

**Figure 2:**
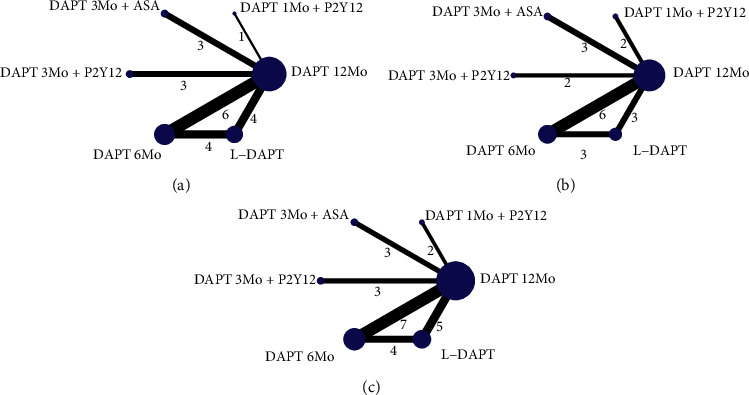
Network plot of treatment comparisons. Network diagram nodes represent individual interventions and nodes connected by lines indicate that these two interventions have been directly compared in a randomized trial. The nodes are weighted by the number of randomized trials evaluating this treatment and lines are weighted by the number of randomized trials evaluating this treatment comparison. (a) Cardiac death. (b) Net adverse clinical events. (c) All-cause mortality, myocardial infarction, major bleeding, any bleeding and definite or probable stent thrombosis.

Supplementary Tables S3–S6 demonstrate the fundamental features of the enrolled studies, baseline characteristics of participants, definitions of endpoints, and outcomes of enrolled trials.  Figure 2 illustrates the network plots. Generally, five trials compared DAPT 12Mo with L-DAPT (*n* = 20351), seven trials compared DAPT 12Mo with DAPT 6Mo (*n* = 12308), three trials compared DAPT 12Mo with DAPT 3Mo + ASA (*n* = 6696), three trials compared DAPT 12Mo with DAPT 3Mo + P2Y12 (*n* = 13168), four trials compared L-DAPT with DAPT 6Mo (*n* = 9839), and two trials compared DAPT 12Mo with DAPT 1Mo + P2Y12 (*n* = 18977). The median follow-up duration was 18 months, with an interquartile range of 12 to 24 months. The distribution of baseline characteristics of patients was balanced across comparisons.

A majority of RCTs were in the low-risk category according to the Cochrane Risk of Bias Tool (Supplementary Table S7). Of the 24 included studies, 23 had a low risk of selection bias for sequence generation, 19 had a low risk of selection bias for allocation concealment, 23 had a low risk of attrition bias, and 24 had a low risk of reporting bias. Minority studies had a high risk of performance bias (3/24, 12.5%) and detection bias (3/24, 12.5%). A majority of studies were in the unclear risk categories of other sources of bias (17/24, 70.8%).

### 3.3. Outcomes of Network Meta-Analysis

#### 3.3.1. All-Cause Mortality and Cardiac Death

We synthesized 24 studies reporting all-cause mortality and 21 studies with a total of 60112 participants reporting cardiac death. Although DAPT 3Mo + P2Y12 was relatively low in all-cause mortality and cardiac death risk than other strategies, there were no significant statistical differences in comparison with other regimes (Supplementary Table S8).

#### 3.3.2. Hemorrhagic Endpoints

In comparison with L-DAPT, DAPT 6Mo, DAPT 3Mo + P2Y12, DAPT 3Mo + ASA, and DAPT 1Mo + P2Y12 were associated with lower risks of major bleeding (Table 1). Simultaneously, L-DAPT increased the risk of any bleeding when compared with other strategies (Table 1). Compared with DAPT 12Mo, DAPT 3Mo + P2Y12 (OR: 0.58, 95% CI: 0.38–0.88) was associated with a reduced risk of any bleeding. Similar rates of hemorrhagic endpoints were noted between DAPT 3Mo + P2Y12, DAPT 3Mo + ASA, and DAPT 1Mo + P2Y12 (Table 1).

#### 3.3.3. Ischemic Endpoints

DAPT 6Mo (OR: 1.88, 95% CI: 1.02–3.40) was associated with a higher risk of definite or probable ST when compared with L-DAPT. In comparison with L-DAPT, DAPT 6Mo (OR: 1.63, 95% CI: 1.15–2.34) and DAPT 12Mo (OR: 1.47, 95% CI: 1.06–1.95) increased the risk of MI. In terms of ischemic endpoints, DAPT 3Mo + P2Y12, DAPT 3Mo + ASA, and DAPT 1Mo + P2Y12 showed no significant difference when compared with L-DAPT (Table 1).

#### 3.3.4. NACE

In comparison with DAPT 3Mo + P2Y12, DAPT 6Mo (OR: 1.42, 95% CI: 1.01–2.04) and DAPT 12Mo (OR: 1.40, 95% CI: 1.05–1.87) were associated with a higher risk of NACE (Supplementary Table S8).

**Figure 3:**
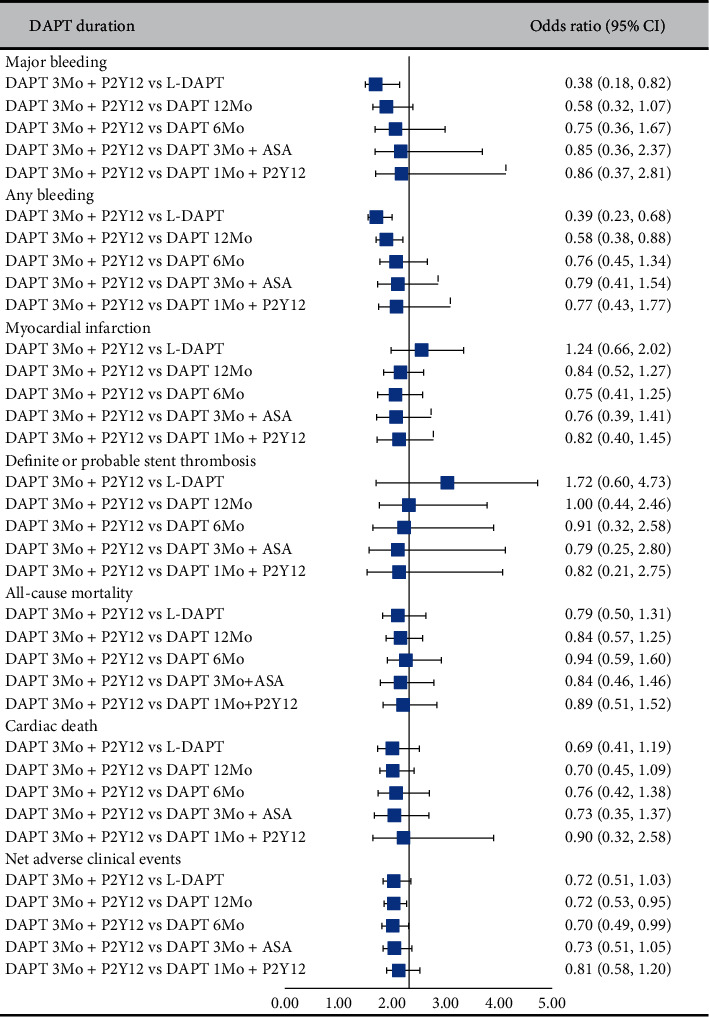
Forest plots. L-DAPT refers to longer-term (>12 months) DAPT, DAPT 12Mo refers to 12-month DAPT, DAPT 6Mo refers to 6-month DAPT, DAPT 3Mo + ASA refers to 3-month DAPT followed by aspirin monotherapy, DAPT 3Mo + P2Y12 refers to 3-month DAPT followed by P2Y12 receptor inhibitor monotherapy, and DAPT 1Mo + P2Y12 refers to 1-month DAPT followed by P2Y12 receptor inhibitor monotherapy. DAPT indicates dual antiplatelet therapy.

Figure 3 illustrates the pooled estimates of different DAPT strategies compared with DAPT 3Mo + P2Y12. Overall, DAPT 3Mo + P2Y12 might be preferred to pursue a trade-off between hemorrhagic and ischemic harm. No significant benefit of ADPT 3Mo + P2Y12 was confirmed when compared with DAPT 3Mo + ASA and DAPT 1Mo + P2Y12. The results should be carefully interpreted because current analyses seem to have scarce “network” to obtain robust results regarding the comparison of DAPT duration.

### 3.4. Rank Probabilities

**Figure 4:**
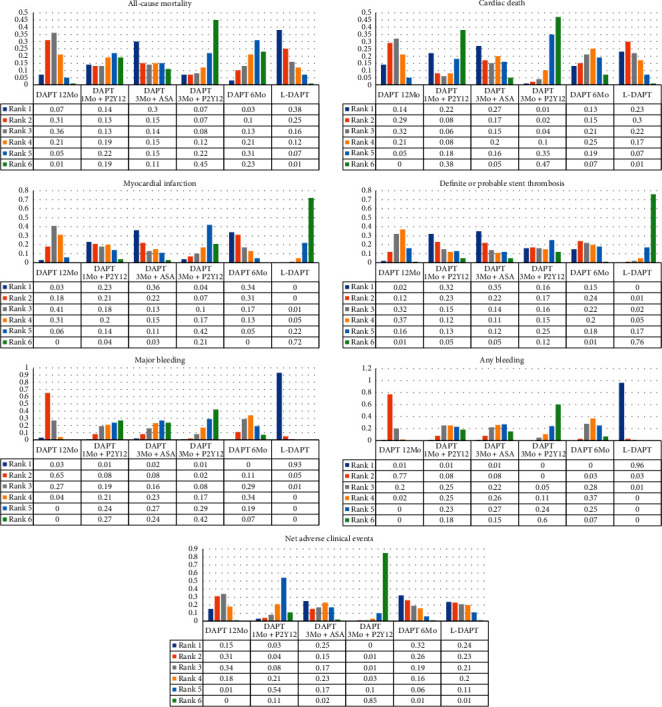
Rankograms for each endpoint. Rank 1 is the worst and rank 6 is best. L-DAPT refers to longer-term (>12 months) DAPT, DAPT 12Mo refers to 12-month DAPT, DAPT 6Mo refers to 6-month DAPT, DAPT 3Mo + ASA refers to 3-month DAPT followed by aspirin monotherapy, DAPT 3Mo + P2Y12 refers to 3-month DAPT followed by P2Y12 receptor inhibitor monotherapy, and DAPT 1Mo + P2Y12 refers to 1-month DAPT followed by P2Y12 receptor inhibitor monotherapy. DAPT indicates dual antiplatelet therapy. (a) All-cause mortality. (b) Cardiac death. (c) Myocardial infarction. (d) Definite or probable stent thrombosis. (e) Major bleeding. (f) Any bleeding. (g) Net adverse clinical events.

The rank probabilities were in accordance with the pooled results quantified by OR (Figure 4). DAPT 3Mo + P2Y12 ranked the best therapy for reducing all-cause mortality, cardiac death, major bleeding, any bleeding, and NACE. L-DAPT ranked first for reducing MI and definite or probable ST, but the least effective in limiting all-cause mortality, major bleeding, and any bleeding.

### 3.5. Network Coherence

The node-splitting analysis confirmed there was no significant difference between direct and indirect effects in closed loops (*p* value >0.05), which verified coherence in all endpoints (Supplementary Table S9). Funnel plots suggested that there was no significant publication bias (Supplementary Figure S2)

### 3.6. Sensitivity Analysis

The SMART-DATE trial, which defined the duration of long-term DAPT as 12 months or longer, might weaken the contradistinction among different interventions. We excluded it and restricted the duration of long-term DAPT to 18 months or longer.  Table 2 shows the pooled results with more significant differences in major bleeding and any bleeding. These outcomes confirmed that the risk of hemorrhagic endpoints increased synchronously when the duration of DAPT was prolonged. In comparison with ≥18 months of DAPT, DAPT 6Mo (OR: 1.53, 95% CI: 1.04–2.17) and DAPT 12Mo (OR: 1.45, 95% CI: 1.01–1.93) increased the risk of MI (Supplementary Table S10).

### 3.7. Subgroup Analysis

We conducted subgroup analyses among patients with ACS for their higher ischemic risk relative to those with stable CAD, and among patients after newer-generation DES implantation owning to its superiority in ischemic outcomes [36, 37].

Results of the participants with ACS and the published sub-analyses are summarized in Supplementary Table S11 [5, 17, 19, 20, 26, 38–43]. Eleven trials with a total of 29863 participants enrolled. When compared with L-DAPT, DAPT 12Mo increased the risk of MI (OR: 2.03, 95% CI: 1.02–3.66) and definite or probable ST (OR: 2.51, 95% CI: 1.05–6.12), whereas other durations showed no significant difference. DAPT 3Mo + P2Y12 decreased the risk of hemorrhagic endpoints when compared with L-DAPT (Supplementary Table S12). DAPT 6Mo (OR: 0.51, 95%: 0.30–0.80) was associated with a lower risk of any bleeding when compared with L-DAPT. Unlike the original meta-analysis, there was no significant benefit of DAPT 3Mo + ASA and DAPT 1Mo + P2Y12 concerning major and any bleeding when compared with L-DAPT. Besides, there were no significant differences between all DAPT strategies in terms of all-cause mortality and NACE (Supplementary Table S12).

Eighteen trials (*n* = 58228) were included in the sub-analysis concerning newer-generation DES (Supplementary Table S13) [5–9, 17–22, 24, 26, 29, 33, 35, 44, 45]. DAPT 6Mo (OR: 0.49, 95% CI: 0.20–0.99), DAPT 3Mo + P2Y12 (OR: 0.27, 95% CI: 0.09–0.82), and DAPT 1Mo + P2Y12 (OR: 0.30, 95% CI: 0.07–0.87) were associated with a lower risk of major bleeding when compared with L-DAPT. What is more, L-DAPT increased the risk of any bleeding when compared with DAPT 6Mo and DAPT 3Mo + P2Y12 (Supplementary Table S14). DAPT 3Mo + P2Y12 was superior in terms of NACE and any bleeding in comparison with DAPT 12Mo. DAPT 6Mo (OR: 1.59, 95% CI: 1.07–2.37) increased the risk of MI when compared with L-DAPT. However, no statistical difference was found between DAPT 12Mo and L-DAPT (Supplementary Table S14).

## 4. Discussion

We conducted a Bayesian network meta-analysis, which included 24 RCTs with 81339 participants, to synthetically evaluate the efficacy and safety of six therapeutic strategies of DAPT in patients undergoing PCI with DESs.

The principal findings were summarized as follows. (1) In comparison with DAPT 12Mo, DAPT 3Mo + P2Y12 decreased the risk of any bleeding and NACE, and it was noninferior for ischemic endpoints, mortality, and major bleeding. (2) 3Mo + P2Y12 might be a reasonable trade-off in terms of reducing bleeding without increasing ischemic harm. (3) DAPT 6Mo, DAPT 3Mo + ASA, and DAPT 1Mo + P2Y12 were comparable to DAPT 12Mo. (4) L-DAPT decreased the risk of MI and definite or probable stent ST. However, L-DAPT led to a higher risk of hemorrhagic events, and the discrimination enlarged when restricting long-term DAPT to 18 months or longer. (5) In the ACS subgroup, DAPT 6Mo was comparable to DAPT 12Mo.

DAPT is the present standard of care after PCI to reduce the risk of recurrent thrombotic events. The DAPT-STEMI trial, the REDUCE trial, and successive meta-analyses proved the feasibility of DAPT 6Mo for those with ACS [17, 19, 46]. However, advanced pharmacological therapies and newer-generation DESs decreased the risk of stent thrombosis while highlighting the hemorrhagic harm of long-term DAPT. Post-discharge bleeding was confirmed to be a contributing factor to cardiac and all-cause mortality, which was more significant than that associated with post-discharge MI [47]. It indicated that the decrease in ischemic risk was more pronounced than that of bleeding risk. Thus, recent trials committed to minimizing bleeding and maximizing the antithrombotic effects by P2Y12 inhibitor monotherapy after a short course of DAPT.

The 2017 ESC guidelines recommended 6-month DAPT in patients with stable CAD and 3-month DAPT in those at high bleeding risk [2]. In contradiction with current guidelines, our study revealed that short-term (1–3 months) DAPT was noninferiority to 6-month DAPT for patients with different clinical presentations and stent types. DAPT 6Mo and short-term (1–3 months) DAPT reduced the risk of any bleeding to a similar degree, whereas only DAPT 3Mo + P2Y12 achieved a significant reduction in comparison with DAPT 12Mo. DAPT 3Mo + P2Y12, which ranked the highest possibilities in the aspect of mortality, bleeding events, and NCAE, might be favored for reducing bleeding and cardiovascular outcomes. Three trials (*n* = 13168) explored the benefit of DAPT 3Mo + P2Y12 and all bleeding events were reduced with a favorable trend to lower ischemic harm [5, 7, 8]. Among them, the TWILIGHT trial evaluated the efficacy of ticagrelor monotherapy concerning clinically relevant bleeding in patients with high bleeding or ischemic risk [7]. The risk of Bleeding Academic Research Consortium (BARC) 3 or 5 bleeding was reduced by half with a slightly increased rate of ST [7]. However, this trial was characterized by the circumstance that it excluded patients with ST-elevation MI [7]. It was not favorable for the composite endpoint of all-cause mortality, non-fatal MI, or non-fatal stroke [7]. As for the DAPT 1Mo + P2Y12 arm, the absence of adjudication of clinical events and withdrawal of ticagrelor monotherapy (24% of participants in the experimental group) in the GLOBAL LEADERS trial might explain the impaired reduction of hemorrhagic risk in it.

Subgroup analysis focusing on the ACS population favored DAPT 3Mo + P2Y12, despite 2020 ESC guidelines recommending 12-month DAPT. This conclusion incorporated the data from the TICO trial and post hoc ACS sub-analysis of the TWILIGHT trial [5, 43]. The TICO trial confirmed that ticagrelor monotherapy following 3-month DAPT, when compared with 12-month ticagrelor-based DAPT, resulted in a modest but statistically significant reduction in a composite outcome of major bleeding and cardiovascular events [5]. However, the absence of patients with high bleeding risk and lower event rates than anticipated might weaken the strength of conclusions. In our study, there was no sufficient evidence to guarantee better efficacy of DAPT 3Mo + P2Y12 in comparison with DAPT 6Mo, especially in patients at high thrombotic risk. Some current studies also favored prolonged DAPT among patients at high ischemic risk, such as those with ACS, diabetes, and previous stenting [48, 49]. In terms of DAPT 3Mo + P2Y12, it was still essential to note that there was a potential for increased ischemic risk.

In our meta-analysis, L-DAPT was superior to other strategies in reducing the risk of MI and definite or probable ST. However, it should be carefully interpreted. First, patients with high bleeding risk or with recent bleeding complications were excluded in a majority of trials, which may weaken the universality of this conclusion. Second, in the newer-generation DESs subgroup, the risk of major bleeding with L-DAPT further increased when compared with the majority of durations. It indicated that L-DAPT should apply with caution under the condition of newer-generation DESs. Third, clopidogrel was predominantly used in the L-DAPT arm. The increased bleeding risk of ticagrelor in comparison with clopidogrel might offset the benefits provided by the P2Y12 inhibitor monotherapy in reducing hemorrhagic events [50]. Generally, L-DAPT reduced the incidence of thrombotic complications at the cost of increased bleeding risk and might only be favored in patients at high thrombotic risk without bleeding concerns.

Previous pair-wise meta-analyses investigated the safety and efficacy of different antithrombotic therapies in patients with atrial fibrillation following PCI [51–54]. They compared dual antithrombotic therapy including a direct oral anticoagulant and an antiplatelet agent with triple antithrombotic therapy including a vitamin K antagonist and DAPT [51–54]. Different from them, our research was committed to different participants and treatments [51–54]. Unlike a prior study by Khan et al., our meta-analysis enrolled more patients with ACS and stratified the short-term DAPT to clarify the safety and efficacy of ≤3 months regimens [55]. We confirmed that short-term (1–3 months) DAPT might be considered in patients after DESs implantation. In patients with ACS, the duration of DAPT should be extended to at least 6 months but might be shorter than 12 months. DAPT 3Mo + P2Y12, which balances thrombotic with hemorrhagic risk, might be preferred in ACS with high bleeding risk. L-DAPT was recommended in patients at high thrombotic risk without bleeding concerns.

More potent P2Y12 inhibitors, low thrombogenicity of newer-generation stents, and optimal procedural techniques might further enable the de-escalation of DAPT. Early de-escalation of DAPT to a P2Y12 inhibitor monotherapy may decrease the hemorrhagic risk attributed to aspirin without deficiency of antithrombotic power [56]. We do believe that bleeding events substantially affect clinical outcomes, medical expenses, and treatment compliance [57]. However, the former trials failed to conduct a head-to-head comparison between a P2Y12 inhibitor monotherapy and aspirin monotherapy. We do not know if aspirin would lead to similar outcomes when early interrupting P2Y12 inhibitors and continuing aspirin. And a recent study confirmed a prasugrel-based low dose strategy from 1 month reducing the risk of bleeding without an increase in ischemia among patients with ACS [58]. Thus, aspirin remains a normative therapy for secondary prevention recommended by current guidelines. It should be acknowledged that short-term DAPT with a P2Y12 inhibitor monotherapy offers a favorable option for balancing bleeding with ischemia in patients with high thrombotic and hemorrhagic risks. Nevertheless, considering the limited RCTs up to now, we should still be cautious not to overgeneralize our results to a larger population.

Our study suffers from several limitations. First, a large majority of eligible RCTs pooled in our meta-analysis excluded patients related to recurrent ischemia and bleeding events. Clopidogrel was predominantly used in enrolled trials. Complex anatomical conditions such as bifurcations, multi-lesions treatment, and left main intervention only represented minority cases. They might weaken the universality of our conclusions. Second, a majority of trials were designed to test the noninferiority of different DAPT strategies. Third, heterogeneity could be witnessed among the enrolled trials in terms of design and endpoint definitions. Fourth, results for ACS patients relied on subgroup or post hoc analyses of the original RCTs, so the results should apply modestly in these patients. Fifth, many trials randomly assigned patients at the time of PCI, which might lower the rates of ischemic events and in favor of patients with higher thrombotic risk. Finally, the comparison between short-term (1–3 months) DAPT was merely based on indirect evidence. On account of limited statistical power, the conclusions must be interpreted with caution.

## 5. Conclusion

Short-term (1–3 months) DAPT is noninferior to DAPT 6Mo in patients with CAD after DESs implantation, while the duration of DAPT should be extended to at least 6 months in patients with ACS. Early (3 months) de-escalation of DAPT to P2Y12 inhibitor monotherapy may be a reasonable option for balancing thrombotic with hemorrhagic risk, especially in those with high risk of bleeding accompanied by ischemia. Long-term (>12 months) DAPT reduces the incidence of thrombotic complications at the cost of increased bleeding risk.

## Figures and Tables

**Table 1:** Pooled estimates of the network meta-analysis of ischemic and hemorrhagic endpoints.

Major bleeding					
DAPT 12Mo	0.67 (0.26, 1.23)	0.67 (0.31, 1.31)	0.58 (0.32, 1.07)	0.77 (0.46, 1.23)	1.50 (0.95, 2.54)
1.49 (0.81, 3.92)	DAPT 1Mo + P2Y12	1.01 (0.39, 3.15)	0.86 (0.37, 2.81)	1.14 (0.52, 3.39)	2.22 (1.07, 7.12)
1.49 (0.76, 3.27)	0.99 (0.32, 2.59)	DAPT 3Mo + ASA	0.85 (0.36, 2.37)	1.15 (0.50, 2.86)	2.26 (1.02, 5.82)
1.74 (0.94, 3.11)	1.16 (0.36, 2.68)	1.18 (0.42, 2.77)	DAPT 3Mo + P2Y12	1.33 (0.60, 2.75)	2.62 (1.21, 5.63)
1.31 (0.81, 2.17)	0.87 (0.29, 1.91)	0.87 (0.35, 2.01)	0.75 (0.36, 1.67)	DAPT 6Mo	1.98 (1.21, 3.50)
0.67 (0.39, 1.05)	0.45 (0.14, 0.93)	0.44 (0.17, 0.98)	0.38 (0.18, 0.82)	0.50 (0.29, 0.83)	L-DAPT

Any bleeding					
DAPT 12Mo	0.74 (0.38, 1.18)	0.74 (0.43, 1.22)	0.58 (0.38, 0.88)	0.76 (0.52, 1.04)	1.48 (1.02, 2.03)
1.35 (0.85, 2.65)	DAPT 1Mo + P2Y12	1.00 (0.51, 2.29)	0.77 (0.43, 1.77)	1.02 (0.58, 2.11)	1.99 (1.13, 4.14)
1.35 (0.82, 2.30)	1.00 (0.44, 1.95)	DAPT 3Mo + ASA	0.79 (0.41, 1.54)	1.03 (0.56, 1.88)	2.01 (1.07, 3.67)
1.73 (1.14, 2.63)	1.29 (0.57, 2.32)	1.27 (0.65, 2.44)	DAPT 3Mo + P2Y12	1.31 (0.75, 2.20)	2.56 (1.46, 4.29)
1.32 (0.96, 1.91)	0.98 (0.47, 1.71)	0.97 (0.53, 1.80)	0.76 (0.45, 1.34)	DAPT 6Mo	1.95 (1.37, 2.74)
0.68 (0.49, 0.98)	0.50 (0.24, 0.89)	0.50 (0.27, 0.94)	0.39 (0.23, 0.68)	0.51 (0.37, 0.73)	L-DAPT

Definite or probable stent thrombosis					
DAPT 12Mo	1.20 (0.54, 3.65)	1.25 (0.54, 2.95)	1.00 (0.44, 2.46)	1.09 (0.62, 2.08)	0.58 (0.34, 1.11)
0.83 (0.27, 1.85)	DAPT 1Mo + P2Y12	1.03 (0.25, 3.28)	0.82 (0.21, 2.75)	0.89 (0.28, 2.53)	0.47 (0.15, 1.35)
0.80 (0.34, 1.87)	0.97 (0.30, 3.96)	DAPT 3Mo + ASA	0.79 (0.25, 2.80)	0.87 (0.32, 2.55)	0.46 (0.18, 1.38)
1.00 (0.41, 2.29)	1.23 (0.36, 4.73)	1.26 (0.36, 4.05)	DAPT 3Mo + P2Y12	1.10 (0.39, 3.10)	0.58 (0.21, 1.67)
0.92 (0.48, 1.62)	1.12 (0.39, 3.58)	1.15 (0.39, 3.13)	0.91 (0.32, 2.58)	DAPT 6Mo	0.53 (0.29, 0.98)
1.73 (0.90, 2.91)	2.11 (0.74, 6.59)	2.16 (0.72, 5.66)	1.72 (0.60, 4.73)	1.88 (1.02, 3.40)	L-DAPT

Myocardial infarction					
DAPT 12Mo	1.03 (0.66, 1.70)	1.10 (0.69, 1.75)	0.84 (0.52, 1.27)	1.12 (0.82, 1.57)	0.68 (0.51, 0.95)
0.97 (0.59, 1.51)	DAPT 1Mo + P2Y12	1.07 (0.53, 2.04)	0.82 (0.40, 1.45)	1.08 (0.63, 1.92)	0.66 (0.38, 1.17)
0.91 (0.57, 1.46)	0.93 (0.49, 1.90)	DAPT 3Mo + ASA	0.76 (0.39, 1.41)	1.01 (0.59, 1.84)	0.62 (0.36, 1.12)
1.19 (0.79, 1.93)	1.22 (0.69, 2.51)	1.32 (0.71, 2.56)	DAPT 3Mo + P2Y12	1.33 (0.80, 2.45)	0.81 (0.50, 1.51)
0.89 (0.64, 1.22)	0.93 (0.52, 1.60)	0.99 (0.54, 1.71)	0.75 (0.41, 1.25)	DAPT 6Mo	0.61 (0.43, 0.87)
1.47 (1.06, 1.95)	1.52 (0.86, 2.62)	1.62 (0.89, 2.78)	1.24 (0.66, 2.02)	1.63 (1.15, 2.34)	L-DAPT

Results in the upper triangle are odds ratios with 95% confidence intervals from the network meta-analysis between the column defining intervention and row defining intervention. Significant results are in bold. L-DAPT refers to longer-term (>12 months) DAPT, DAPT 12Mo refers to 12-month DAPT, DAPT 6Mo refers to 6-month DAPT, DAPT 3Mo + ASA refers to 3-month DAPT followed by aspirin monotherapy, DAPT 3Mo + P2Y12 refers to 3-month DAPT followed by a P2Y12 receptor inhibitor monotherapy, and DAPT 1Mo + P2Y12 refers to 1-month DAPT followed by a P2Y12 receptor inhibitor monotherapy. DAPT indicates dual antiplatelet therapy.

**Table 2:** Pooled estimates of hemorrhagic endpoints of sensitivity analysis.

Major bleeding					
DAPT 12Mo	0.65 (0.24, 1.29)	0.67 (0.31, 1.37)	0.58 (0.31, 1.13)	0.76 (0.44, 1.25)	1.53 (0.95, 2.75)
1.54 (0.77, 4.24)	DAPT 1Mo + P2Y12	1.03 (0.37, 3.63)	0.89 (0.36, 3.08)	1.18 (0.49, 3.53)	2.38 (1.05, 8.20)
1.50 (0.73, 3.26)	0.97 (0.28, 2.67)	DAPT 3Mo + ASA	0.86 (0.34, 2.41)	1.13 (0.46, 2.90)	2.30 (1.00, 6.28)
1.73 (0.89, 3.27)	1.12 (0.32, 2.78)	1.16 (0.41, 2.93)	DAPT 3Mo + P2Y12	1.31 (0.54, 2.88)	2.66 (1.17, 6.36)
1.32 (0.80, 2.27)	0.85 (0.28, 2.02)	0.89 (0.35, 2.18)	0.76 (0.35, 1.85)	DAPT 6Mo	2.04 (1.17, 3.93)
0.65 (0.36, 1.05)	0.42 (0.12, 0.95)	0.44 (0.16, 1.00)	0.38 (0.16, 0.85)	0.49 (0.25, 0.85)	L-DAPT

Any bleeding					
DAPT 12Mo	0.75 (0.37, 1.16)	0.73 (0.43, 1.21)	0.58 (0.37, 0.87)	0.72 (0.50, 1.00)	1.53 (1.05, 2.12)
1.33 (0.87, 2.72)	DAPT 1Mo + P2Y12	0.98 (0.50, 2.40)	0.76 (0.43, 1.79)	0.97 (0.55, 2.10)	2.02 (1.18, 4.33)
1.37 (0.82, 2.31)	1.02 (0.42, 1.99)	DAPT 3Mo + ASA	0.79 (0.41, 1.52)	0.99 (0.53, 1.84)	2.10 (1.09, 3.84)
1.74 (1.15, 2.70)	1.32 (0.56, 2.33)	1.27 (0.66, 2.45)	DAPT 3Mo + P2Y12	1.26 (0.72, 2.16)	2.67 (1.50, 4.52)
1.39 (1.00, 1.99)	1.04 (0.48, 1.80)	1.01 (0.54, 1.90)	0.79 (0.46, 1.39)	DAPT 6Mo	2.10 (1.42, 3.10)
0.65 (0.47, 0.96)	0.50 (0.23, 0.85)	0.48 (0.26, 0.91)	0.37 (0.22, 0.67)	0.48 (0.32, 0.71)	L-DAPT

Results in the upper triangle are odds ratios with 95% confidence intervals from the network meta-analysis between the column defining intervention and row defining intervention. Significant results are in bold. L-DAPT refers to longer-term (>18 months) DAPT, DAPT 12Mo refers to 12-month DAPT, DAPT 6Mo refers to 6-month DAPT, DAPT 3Mo + ASA refers to 3-month DAPT followed by aspirin monotherapy, DAPT 3Mo + P2Y12 refers to 3-month DAPT followed by a P2Y12 receptor inhibitor monotherapy, and DAPT 1Mo + P2Y12 refers to 1-month DAPT followed by a P2Y12 receptor inhibitor monotherapy. DAPT indicates dual antiplatelet therapy.

## Data Availability

The datasets used or analyzed during the current study are available in the appendix, and more data are available from the corresponding author on reasonable request.

## Abbreviations

ACC: American College of Cardiology
ACS: Acute coronary syndrome
ADDIS: Aggregate data drug information system
AHA: American Heart Association
BARC: Bleeding Academic Research Consortium
BMS: Bare-metal stent
CAD: Coronary artery disease
CI: Confidence interval
DAPT: Dual antiplatelet therapy
DAPT-STEMI: Six-month versus 12-month dual antiplatelet therapy after drug-eluting stent implantation in ST-elevation myocardial infarction (DAPT-STEMI): randomized, multicentre, noninferiority trial
DAPT 6Mo: 6-month dual antiplatelet therapy
DAPT 12Mo: 12-month dual antiplatelet therapy
DES: Drug-eluting stent
ESC: European Society of Cardiology
L-DAPT: Long-term dual antiplatelet therapy
MCMC: Markov chain Monte Carlo
MI: Myocardial infarction
NACE: Net adverse clinical events
OR: Odds ratio
PCI: Percutaneous coronary intervention
PRISMA: Preferred Reporting Items for Systematic Reviews and Meta-Analyses
PSRF: The Potential Scale Reduction Factor
RCT: Randomized controlled trial
REDUCE: Final results of the randomized evaluation of short-term dual antiplatelet therapy in patients with acute coronary syndrome treated with a new-generation stent
DAPT 3Mo + ASA: 3-month DAPT followed by aspirin monotherapy
DAPT 3Mo + P2Y12: 3-month DAPT followed by P2Y12 receptor inhibitor monotherapy
DAPT 1Mo + P2Y12: 1-month DAPT followed by P2Y12 receptor inhibitor monotherapy
GLOBAL LEADERS: Ticagrelor plus aspirin for 1 month, followed by ticagrelor monotherapy for 23 months vs. aspirin plus clopidogrel or ticagrelor for 12 months, followed by aspirin monotherapy for 12 months after implantation of a drug-eluting stent: a multicentre, open-label, randomized superiority trial
ST: Stent thrombosis
TICO: Effect of ticagrelor monotherapy vs. ticagrelor with aspirin on major bleeding and cardiovascular events in patients with acute coronary syndrome: the TICO randomized clinical trial
TWILIGHT: Ticagrelor with aspirin or alone in high-risk patients after coronary intervention.

## Supplementary Materials

Supplementary Materials
*Supplementary Table S1.* PRISMA checklist; *Supplementary Table S2*. Individual search algorithm; *Supplementary Table S3*. Baseline characteristics of the included trials; *Supplementary Table S4*. Baseline characteristics of participants; *Supplementary Table S5.* Definitions of clinical endpoints; *Supplementary Table S6.* The number of participants who had each outcome; *Supplementary Table S7.* Risk of bias of included trials; *Supplementary Table S8*. Pooled estimates of the network meta-analysis; *Supplementary Table S9.* Node-splitting analysis of inconsistency; *Supplementary Table S10*. Pooled estimates of sensitivity analysis; *Supplementary Table S11.* The number of participants with acute coronary syndrome; *Supplementary Table S12*. Pooled estimates of the network meta-analysis with acute coronary syndrome; *Supplementary Table S13*. The number of participants with newer-generation drug-eluting stents; *Supplementary Table S14.* Pooled estimates of the network meta-analysis with newer-generation drug-eluting stents; *Supplementary Figure S1.* Pair-wise meta-analysis of all endpoints; *Supplementary Figure S2.* Funnel plots.
