# Supplementary material for: Efficacy and Safety of Dual Antiplatelet Therapy in Patients Undergoing Coronary Stent Implantation: A Systematic Review and Network Meta-Analysis
Source: J Interv Cardiol. 2021 May 5;2021:9934535. doi: 10.1155/2021/9934535 (PMC8118746; doi:10.1155/2021/9934535)

Efficacy and safety of dual antiplatelet therapy in patients undergoing coronary stent implantation: A systematic review and network meta-analysis

JOURNAL OF INTERVENTIONAL CARDIOLOGY

Yi Xu [1], Yimin Shen [1], Delong Chen [1], Pengfei Zhao [2,3], Jun Jiang [1]*

[1] Department of Cardiology, The Second Affiliated Hospital of Zhejiang University School of Medicine, 88 Jiefang Road, Hangzhou, Zhejiang 310009, China

[2] School of Chinese Materia Medica, Nanjing University of Chinese Medicine, 138 Xianlin Avenue, Nanjing 210023, China

[3] State Key Laboratory of Drug Research, Shanghai Institute of Materia Medica, Chinese Academy of Sciences, 501 Haike Road, Shanghai 201203, China

* Correspondence: jiang-jun@zju.edu.cn

Supplementary Material

Table of Contents

Supplementary Table S1 PRISMA checklist 4

Supplementary Table S2 Individual search algorithm 8

Supplementary Table S3 Baseline characteristics of the included trials 11

Supplementary Table S4 Baseline characteristics of participants 18

Supplementary Table S5 Definitions of clinical endpoints of the included trials 24

Supplementary Table S6 The number of participants who had each outcome in each study 31

Supplementary Table S7 Risk of bias of included trials using the Cochrane Risk Assessment Tool 34

Supplementary Table S8 Pooled estimates of the network meta-analysis of each endpoint. 36

Supplementary Table S9 Node-splitting analysis of Inconsistency for all endpoints 39

Supplementary Table S10 Pooled estimates of the network meta-analysis of sensitivity analysis 41

Supplementary Table S11 The number of participants with acute coronary syndrome who had each outcome 44

Supplementary Table S12 Pooled estimates of the network meta-analysis of each endpoint in participants with acute coronary syndrome 46

Supplementary Table S13 The number of participants with newer-generation drug-eluting stents who had each outcome 48

Supplementary Table S14 Pooled estimates of the network meta-analysis of each endpoint in Participants with newer-generation drug-eluting stents 50

Supplementary Fig. S1 Pair-wise meta-analysis of all endpoints 52

Supplementary Fig. S2 Funnel plots of each endpoint. 59

Supplementary Table S1 PRISMA checklist

Section/topic	#	Checklist item	Reported on page #
**TITLE**			
Title	1	Identify the report as a systematic review incorporating a network meta-analysis (or related form of meta-analysis).	1
**ABSTRACT**			
Structured summary	2	Provide a structured summary including, as applicable: 1) Background: main objectives; 2)Methods: data sources; study eligibility criteria, participants, and interventions; study appraisal; and synthesis methods, such as network meta-analysis; 3) Results: number of studies and participants identified; summary estimates with corresponding confidence/credible intervals; treatment rankings may also be discussed. Authors may choose to summarize pairwise comparisons against a chosen treatment included in their analyses for brevity. 4) Discussion/Conclusions: limitations; conclusions and implications of findings. 5) Other: primary source of funding; systematic review registration number with registry name.	2
**INTRODUCTION**			
Rationale	3	Describe the rationale for the review in the context of what is already known, including mention of why a network meta-analysis has been conducted.	3
Objectives	4	Provide an explicit statement of questions being addressed with reference to participants, interventions, comparisons, outcomes, and study design (PICOS).	3
**METHODS**			
Protocol and registration	5	Indicate if a review protocol exists, if and where it can be accessed (e.g., Web address), and, if available, provide registration information including registration number.	N/A

Eligibility criteria	6	Specify study characteristics (e.g., PICOS, length of follow-up) and report characteristics (e.g., years considered, language, publication status) used as criteria for eligibility, giving rationale. Clearly describe eligible treatments included in the treatment network, and note whether any have been clustered or merged into the same node (with justification).	4
Information sources	7	Describe all information sources (e.g., databases with dates of coverage, contact with study authors to identify additional studies) in the search and date last searched.	4
Search	8	Present full electronic search strategy for at least one database, including any limits used, such that it could be repeated.	Appendix Table S2
Study selection	9	State the process for selecting studies (i.e., screening, eligibility, included in systematic review, and, if applicable, included in the meta-analysis).	4
Data collection process	10	Describe method of data extraction from reports (e.g., piloted forms, independently, in duplicate) and any processes for obtaining and confirming data from investigators.	4-5
Data items	11	List and define all variables for which data were sought (e.g., PICOS, funding sources) and any assumptions and simplifications made.	4-5
Geometry of the network	S1	Describe methods used to explore the geometry of the treatment network under study and potential biases related to it. This should include how the evidence base has been graphically summarized for presentation, and what characteristics were compiled and used to describe the evidence base to readers.	5
Risk of bias within individual studies	12	Describe methods used for assessing risk of bias of individual studies (including specification of whether this was done at the study or outcome level), and how this information is to be used in any data synthesis.	5
Summary measures	13	State the principal summary measures (e.g., risk ratio, difference in means). Also describe the use of additional summary measures assessed, such as treatment rankings and surface under the cumulative ranking curve (SUCRA) values, as well as modified approaches used to present summary findings from meta-analyses.	5
Planned method of analysis	14	Describe the methods of handling data and combining results of studies for each network meta-analysis. This should include, but not be limited to: handling of multi-group trials; Selection of variance structure; Selection of prior	5

		distributions in Bayesian analyses; and Assessment of model fit.	
Assessment of inconsistency	S2	Describe the statistical methods used to evaluate the agreement of direct and indirect evidence in the treatment network(s) studied. Describe efforts taken to address its presence when found.	5
Risk of bias across studies	15	Specify any assessment of risk of bias that may affect the cumulative evidence (e.g., publication bias, selective reporting within studies).	5
Additional analyses	16	Describe methods of additional analyses if done, indicating which were pre-specified. This may include, but not be limited to, the following: sensitivity or subgroup analyses; Meta-regression analyses; Alternative formulations of the treatment network; and Use of alternative prior distributions for Bayesian analyses (if applicable).	5
**RESULTS**			
Study selection	17	Give numbers of studies screened, assessed for eligibility, and included in the review, with reasons for exclusions at each stage, ideally with a flow diagram.	6
Presentation of network structure	S3	Provide a network graph of the included studies to enable visualization of the geometry of the treatment network.	Figure 2
Summary of network geometry	S4	Provide a brief overview of characteristics of the treatment network. This may include commentary on the abundance of trials and randomized patients for the different interventions and pairwise comparisons in the network, gaps of evidence in the treatment network, and potential biases reflected by the network structure.	6-7
Study characteristics	18	For each study, present characteristics for which data were extracted (e.g., study size, PICOS, follow-up period) and provide the citations.	Appendix Table S3, S4, and S5
Risk of bias within studies	19	Present data on risk of bias of each study and, if available, any outcome level assessment.	Appendix Table S7
Results of individual studies	20	For all outcomes considered (benefits or harms), present, for each study: 1) simple summary data for each intervention group, and 2) effect estimates and confidence intervals. Modified approaches may be needed to deal with information from larger networks.	Appendix Table S6

Synthesis of results	21	Present results of each meta-analysis done, including confidence/credible intervals. In larger networks, authors may focus on comparisons versus a particular comparator (e.g., placebo or standard care), with full findings presented in an appendix. League tables and forest plots may be considered to summarize pairwise comparisons. If additional summary measures were explored (such as treatment rankings), these should also be presented.	8-10
Exploration of inconsistency	S5	Describe results from investigations of inconsistency. This may include such information as measures of model fit to compare consistency and inconsistency models, P values from statistical tests, or summary of inconsistency estimates from different parts of the treatment network.	11
Risk of bias across studies	22	Present results of any assessment of risk of bias across studies for the evidence base being studied.	11
Results of additional analyses	23	Give results of additional analyses, if done (e.g., sensitivity or subgroup analyses, meta-regression analyses, alternative network geometries studied, alternative choice of prior distributions for Bayesian analyses, and so forth).	11-14
**DISCUSSION**			
Summary of evidence	24	Summarize the main findings, including the strength of evidence for each main outcome; consider their relevance to key groups (e.g., health care providers, researchers, and policymakers).	14-17
Limitations	25	Discuss limitations at study and outcome level (e.g., risk of bias), and at review level (e.g., incomplete retrieval of identified research, reporting bias). Comment on the validity of the assumptions, such as transitivity and consistency. Comment on any concerns regarding network geometry (e.g., avoidance of certain comparisons).	18
Conclusions	26	Provide a general interpretation of the results in the context of other evidence, and implications for future research.	18
**FUNDING**			
Funding	27	Describe sources of funding for the systematic review and other support (e.g., supply of data); role of funders for the systematic review. This should also include information regarding whether funding has been received from manufacturers of treatments in the network and/or whether some of the authors are content experts with professional conflicts of interest that could affect use of treatments in the network.	N/A

Supplementary Table S2 Individual search algorithm

PubMed	search strategies	Results
#1	Drug-eluting stent[MeSH Terms]	11,909
#2	coronary stent*[Title/Abstract]	7,328
#3	#1 OR #2	18,004
#4	Percutaneous Coronary Intervention[MeSH Terms]	55,427
#5	(percutaneous coronary[Title/Abstract]) AND revascular*[Title/Abstract]	9,966
#6	PCI[Title/Abstract]	26,763
#7	#4 OR #5 OR #6	67,755
#8	Platelet Aggregation Inhibitors[MeSH Terms]	37,171
#9	antiplatelet*[Title/Abstract] OR anti-platelet*[Title/Abstract] OR antithrombocytic[Title/Abstract] OR anti-thrombocytic[Title/Abstract] OR cyclooxygenase inhibitor*[Title/Abstract] OR thienopyridine*[Title/Abstract] OR thromboxane A2 inhib*[Title/Abstract] OR aspirin[MeSH Terms] OR clopidogrel[Title/Abstract] OR ticagrelor[Title/Abstract] OR prasugrel[Title/Abstract]	82,739
#10	#8 OR #9	96,044
#11	#3 OR #7 AND #10	10,871
#12	((randomized controlled trial[Publication Type]) OR randomized controlled trial[MeSH Terms])OR controlled clinical trial[Publication Type]	746,412
#13	(((Animal Experimentation[MeSH Terms]) OR Animals,Laboratory[MeSH Terms]) OR Models, Animal[MeSH Terms]) OR rodentia[MeSH Terms]	3,418,355
#14	#11 AND #12 NOT #13	2,480
**Embase**	**search strategies**	**Results**
#1	'drug-eluting stent'/exp	33,827
#2	coronary:ab,ti AND stent*:ab,ti	51,190
#3	#1 OR #2	66,566
#4	percutaneous coronary intervention'/exp	102,108

#5	percutaneous AND coronary AND revascular*:ab,ti	24,709
#6	pci:ab,ti	57,812
#7	#4 OR #5 OR #6	126,939
#8	'antithrombocytic agent'/exp	352,634
#9	antiplatelet*:ab,ti OR 'anti platelet*':ab,ti OR antithrombocytic:ab,ti OR 'anti thrombocytic':ab,ti OR (cyclooxygenase:ab,ti AND inhibitor*:ab,ti) OR thienopyridine*:ab,ti OR (thromboxane:ab,ti AND a2:ab,ti AND inhib*:ab,ti) OR 'aspirin'/exp OR clopidogrel:ab,ti OR ticagrelor:ab,ti OR prasugrel:ab,ti	278,996
#10	#8 OR #9	393,556
#11	andomized controlled trial' OR 'randomized controlled trials as topic'/exp OR random*:ti,ab	1,639,922
#12	'animals, laboratory'/exp OR 'animal experiment'/exp OR 'animal model'/exp OR 'rodent'/exp	4,635,855
#13	(#3 OR #7) AND #10 AND #11 NOT #12	8,341
**Cochrane Library**	**search strategies**	**Results**
#1	[mh "drug-eluting stents"]	1,423
#2	coronary stent*:ti,ab,kw	8527
#3	#1 OR #2	8606
#4	[mh "Percutaneous Coronary Intervention"]	5567
#5	percutaneous coronary revascular*:ti,ab,kw	4204
#6	PCI:ti,ab,kw	8366
#7	#4 OR #5 OR #6	12846
#8	[mh "Platelet aggregation inhibitors"] OR [mh "Cyclooxygenase Inhibitors"] OR [mh Thienopyridines] OR [mh "Thromboxane A2"/AI] OR [mh "Purinergic P2Y Receptor Antagonists"]	6264
#9	antiplatelet* OR anti-platelet* OR antithrombocytic OR anti-thrombocytic OR clopidogrel or ticagrelor or prasugrel OR aspirin*:ti,ab,kw	20109
#10	#8 OR #9	22318
#11	randomized OR clinical trials:ti,ab,kw	1128131
#12	rat OR rats OR mouse OR mice:ti,ab,kw OR [mh animals]	589756
#13	(#3 OR #7)AND #10 AND #11 NOT #12	1698

Web of Science	search strategies	Results
#1	TS=drug-eluting stent	13626
#2	TS=coronary stent*	43772
#3	#1 OR #2	47275
#4	TS=Percutaneous Coronary Intervention	54290
#5	TS=percutaneous coronary revascular*	15203
#6	TS=PCI	35542
#7	#4 OR #5 OR #6	72283
#8	TS=Platelet aggregation inhibitors	9529
#9	TS=(antiplatelet*OR anti-platelet* OR antithrombocytic OR anti-thrombocytic OR cyclooxygenase inhibitor* OR thienopyridine* OR thromboxane A2 inhibitor* OR aspirin OR clopidogrel OR ticagrelor OR prasugrel)	103258
#10	#8 OR #9	110089
#11	TS=(clinical trial* OR controlled trial* OR random* trial* OR random*stud*)	1210556
#12	(#3 OR #7) AND #10 AND #11	3614
#13	(#3 OR #7) AND #10 AND #11 limit article	2678
**ClinicalTrials.gov**		
379 Studies found for: Antiplatelet Drug OR Antiplatelet Aggregation Inhibitors | Interventional Studies | PCI OR Percutaneous Coronary Intervention OR drug-eluting stent		
**Unpublished evidence**		
we used following keywords in different combinations to search unpublished trials at relevant meetings (American Heart Association, American College of Cardiology, European Society of Cardiology): antiplatelet, aspirin, drug eluting stent, percutaneous coronary intervention, from 2018 to 2020.		

Supplementary Table S3 Baseline characteristics of the included trials

Trials	Randomization	Stent type	Follow-up	Primary endpoint	Treatment strategy
**TICO**	At index PCI	BP-SES	12months	Composite of major bleeding and adverse cardiac and cerebrovascular events	DAPT 3Mo+P2Y12: ticagrelor (90 mg twice daily,12months) + aspirin (100 mg daily,3months);<br>DAPT 12Mo: ticagrelor (90 mg twice daily,12months) + aspirin (100 mg daily,12months)
**STOPDAPT-2**	At index PCI	EES	12months	Composite of cardiovascular death, MI, definite ST, ischemic or hemorrhagic stroke, or TIMI major or minor bleeding	DAPT 1Mo+P2Y12: aspirin (81-200mg daily dose,1months) + clopidogrel (75mg daily dose,1months) or prasugrel (3.75mg daily dose,1months) and followed by clopidogrel monotherapy for up to 5 years;<br>DAPT 12Mo: aspirin (81-200mg daily dose,12 months) + clopidogrel (75 mg daily dose,12months) or prasugrel (3.75 mg daily dose,12months) and followed by aspirin monotherapy for up to 5 years

**TWILIGHT**	At 3 months after PCI	DES	12months	The first occurrence of BARC type 2, 3, or 5 bleeding	DAPT 3Mo+P2Y12: ticagrelor (90 mg twice daily) and aspirin (81-100 mg daily) for 3 months. Then switched saspirin to placebo for an additional 12 months along with continuation ticagrelor treatment;<br>DAPT 12Mo: ticagrelor (90 mg twice daily,15months) + aspirin (81-100 mg daily,15 months)
**SMART-CHOICE**	At the index or within 3 months after the index PCI	EES/BP-SES/ZES	12months	Composite of all-cause death, MI, or stroke	DAPT 3Mo+P2Y12: aspirin (100mg daily dose, 3months) + clopidogrel (75 mg daily dose, indefinitely) or prasugrel (10 mg daily dose, indefinitely) or ticagrelor (90 mg twice daily, indefinitely);<br>DAPT 12Mo: Aspirin (100mg daily dose, indefinitely) + clopidogrel (75 mg daily dose, indefinitely) or prasugrel (10 mg daily dose, indefinitely) or ticagrelor (90 mg twice daily, indefinitely)
**REDUCE**	At index PCI	MASCOT	24months	Composite occurrence of all-cause death, MI, ST, stroke, TVR and bleeding	DAPT 3Mo+ASA :Aspirin (indefinitely)+Pras-ugrel (10 mg daily dose,3months) or Ticagrelor (180 mg daily dose,3months) or Clopidogrel (75mg daily dose,3months);<br>DAPT 12Mo: Aspirin (indefinitely)+Prasu-grel (10 mg daily dose,12months) or Ticagrelor (180 mg daily dose,12months) or Clopidogrel (75mg daily dose,12m-onths)

**GLOBAL LEADERS**	At index PCI	BES	24months	Composite of all-cause death or new Q-wave MI	DAPT 1Mo+P2Y12:75-100 mg aspirin daily in combination with 90 mg ticagrelor twice daily for 1 month, followed by 90 mg ticagrelor twice daily for 23 months;<br>DAPT 12Mo:75-100 mg aspirin daily in combination with either 75 mg clopidogrel daily (for patients with stable coronary artery disease) or 90 mg ticagrelor twice daily (for patients with acute coronary syndromes), followed by 75-100 mg aspirin daily for 12 months
**OPTIMA-C**	At index PCI	BES/ZES	12months	Composite of cardiac death, target vessel-related MI, ischemia-driven TLR	DAPT 6Mo: aspirin (100 mg daily dose, indefinitely) + clopidogrel (75 mg daily dose, 6 months) ;<br>DAPT 12Mo: aspirin (100 mg daily dose, indefinitely) + clopidogrel (75 mg daily dose, 12 months)
**DAPT-STEMI**	At 6 months after PCI	BES/EES/ZES	24months	Composite of all-cause mortality, any MI, any revascularization, stroke, major bleeding	DAPT 6Mo: aspirin (75-100mg daily dose, indefinitely) +Prasugrel (10 mg daily dose,6months) or Ticagrelor (90 mg twice daily,6months) or Clopidogrel (75mg daily dose,6months);<br>DAPT 12Mo: aspirin (75-100mg daily dose, indefinitely) + Prasugrel (10 mg daily dose,12months) or Ticagrelor (90 mg twice daily,12months) or Clopidogrel (75mg daily dose,12months)
**SMART-DATE**	At index PCI	EES/ZES/BES	18months	Composite of all-cause mortality, MI or stroke	DAPT 6Mo: aspirin (100mg daily dose, indefinitely) + clopidogrel (75mg daily dose ,6months) or prasugrel (10 mg daily dose ,6months) or ticagrelor (90mg twice per day,6months);<br>L-DAPT: aspirin (100mg daily dose, indefinitely) + clopidogrel (75mg daily dose ,12 months or longer) or

					prasugrel (10 mg daily dose ,12 months or longer) or ticagrelor (90mg twice per day,12 months or longer)
**NIPPON**	At index PCI	BES	18months	Composite of all cause death, Q-wave or non-Q-wave MI, cerebrovascular events, and major bleeding events	L-DAPT: aspirin (81-162mg daily dose, indefinitely) + clopidogrel (75mg daily dose,18 months) /ticlopidine (200mg daily dose,18months);<br>DAPT 6Mo: aspirin (81-162mg daily dose, indefinitely) + clopidogrel (75mg daily dose,6 months) /ticlopidine (200mg daily dose,6 months).
**IVUS-XPL**	At index PCI	EES	12months	Composite of cardiac death, MI, stroke, or TIMI major bleeding	DAPT 6Mo: aspirin (100 mg daily dose, indefinitely) + clopidogrel (75 mg daily dose, 6 months);<br>DAPT 12Mo: aspirin (100 mg daily dose, indefinitely) + clopidogrel (75 mg daily dose, 12 months)
**OPTIDUAL**	12±3 months after PCI	SES/PES/ZES/ EES	36months	Composite of all-cause mortality, non-fatal MI, stroke, or major bleeding	L-DAPT: aspirin (75-160mg daily dose, indefinitely) + clopidogrel (75mg daily dose,48 months);<br>DAPT 12Mo: aspirin (75-160mg daily dose, indefinitely) + clopidogrel (75mg daily dose,12months)
**I-LOVE-IT 2**	At index PCI	BP-SES	18months	Composite of cardiac death, target vessel MI, or clinically indicated TLV	DAPT 6Mo: aspirin (100 mg daily dose, indefinitely) + clopidogrel (75 mg daily dose, 6 months);<br>DAPT 12Mo: aspirin (100 mg daily dose, indefinitely) + clopidogrel (75 mg daily dose, 12 months)
**ISAR-SAFE**	At 6 (-1/+2) months after DES implantation	PES/SES/EES/ ZES/BES/BMS	15months	Composite of death, myocardial infarction, ST (definite or probable), stroke, or thrombolysis in MI major bleeding	DAPT 6Mo: aspirin (81-200mg daily dose, indefinitely) +clopidogrel (75 mg daily dose, 6 months) and clopidogrel was swithched to placebo after 6 months;<br>DAPT 12Mo: aspirin (81-200 mg daily dose, indefinitely) + clopidogrel (75 mg daily dose, 12months)

**ITALIC**	6 months after PCI	EES	24months	Composite of all-cause mortality, MI, TVR, stroke, or major bleeding according to the TIMI criteria	DAPT 6Mo: aspirin (indefinitely) + clopidogrel (75mg daily dose, 6 months) or prasugrel (10mg daily dose, 6 months) or ticagrelor (90mg twice per day, 6months);<br>L-DAPT: aspirin (indefinitely) + clopid-ogrel (75mg daily dose,24 months) or prasugrel (10mg daily dose,24months) or ticagrelor (90mg twice per day,24 months)
**DAPT Study**	12 months after PCI	EES/PES/ZES/ SES	33months	Composite of definite or probable ST and of major adverse cardiovascular and cerebrovascular events	L-DAPT: aspirin (75-162mg daily dose, indefinitely) + clopidogrel (75mg daily dose, 30months) or prasugrel (10 or 5 mg daily dose,30months);<br>DAPT 12Mo: Aspirin (75-162mg daily dose, indefinitely) + clopidogrel (75 mg daily dose, 12 months) or prasugrel (10 or 5mg daily dose, 12 months) continued by placebo
**DES LATE**	12 to 18 months after PCI	SES/PES/ZES/ EES/others	Median length of follow-up was 42.0 months (interquartile range, 24.7-50.7 months)	Composite of death resulting from cardiac causes, MI, or stroke 24 months after randomization.	DAPT 12Mo: aspirin (100-200mg daily dose, indefinitely) + clopidogrel (75mg daily dose,12 months);<br>L-DAPT: aspirin (100-200mg daily dose, indefinitely) + clopidogrel (75mg daily dose, 36 months)
**SECURITY**	At index PCI	BES/EES/ZES	24months	Composite of cardiac death, MI, stroke, definite or probable stent thrombosis, or BARC criteria type 3 or 5 bleeding	DAPT 6Mo: aspirin (indefinitely) + clopidogrel (75 mg daily dose, 6 months);<br>DAPT 12Mo: aspirin (indefinitely) +clopidogrel (75 mg daily dose, 12 months)
**ARCTIC-Interruption**	12 months after PCI	First or second-generation DES	Median duration of follow-up was 17 months (interquartile	Composite of death, myocardial infarction, stent thrombosis, stroke, or urgent revascularisation	L-DAPT: aspirin (indefinitely) + clopidogrel (75-150mg daily dose, 18-30months) or purasugrel (10 mg daily dose, 18-30months);<br>DAPT12Mo: aspirin (indefinitely) + clopidogrel (75-150mg

			range,15 to 18)		daily dose, 12months) or purasugrel (10 mg daily dose, 12months)
**OPTIMIZE**	At index PCI	ZES	12months	Composite of all-cause mortality, MI, stroke, or major bleeding	DAPT 3Mo+ASA: aspirin (100-200mg daily dose, indefinitely) + clopidogrel (75 mg daily dose,3 months); DAPT 12Mo: aspirin (100-200mg daily dose,indefinitely) + clopidogrel (75 mg daily dose, 12 months)
**PRODIGY**	30±5 days after PCI	EES/PES/ZES/ BMS	24months	Composite of death of any cause, MI, or cerebrovascular accident	L-DAPT: aspirin (80-160mg daily dose, indefinitely) + clopidogrel (75 mg daily dose, 24 months); DAPT 6Mo: aspirin (80-160mg daily dose, indefinitely) + clopidogrel (75 mg daily dose, 6 months)
**RESET**	At index PCI	ZES/SES/EES	12months	Composite of death from cardiovascular cause, MI, ST, ischemia-driven TVR, or bleeding	DAPT 3Mo+ASA: aspirin (100mg daily dose, indefinitely) + clopidogrel (75 mg daily dose,3months); DAPT 12Mo: aspirin (100mg daily dose, indefinitely) + clopidogrel (75 mg daily dose,12months)
**EXCELLENT**	At index PCI	EES/SES	12months	Composite of cardiac death, MI, or TVR	DAPT 6Mo: aspirin (100-200mg daily dose, 12months) + clopidogrel (75 mg daily dose,6 months); DAPT12Mo: aspirin (100-200mg daily dose, 12months) + clopidogrel (75 mg daily dose, 12 months)
**REAL-ZEST LATE**	12 months after PCI	SES/PES/ZES/ other	Median duration of follow-up was 19.2 months (interquartile range, 13.2 to 24.1)	The first occurrence of MI or death from cardiac causes after assignment to a treatment group	L-DAPT: aspirin (100-200mg daily dose, indefinitely) + clopidogrel (75 mg daily dose, 36months); DAPT 12Mo: aspirin (100-200mg daily dose, indefinitely) + clopidogrel (75 mg daily dose,12 months)

PCI, percutaneous coronary intervention; MI, myocardial infarction; ST, stent thrombosis; DAPT, dual antiplatelet therapy; L-DAPT, longer than 12 months DAPT; DAPT 12Mo,12

months DAPT; S-DAPT+ASA, shorter than 6-month DAPT followed by aspirin monotherapy; S-DAPT+P2Y12, shorter than 6-month DAPT followed by a P2Y12 receptor inhibitor monotherapy; TIMI, thrombolysis in MI; BARC, Bleeding Academic Research Consortium; BES, biolimus-eluting stent ; ZES, zotarolimus-eluting stent; BP-SES, biodegradable polymer sirolimus-eluting stent; EES, everolimus-eluting stent; PES, paclitaxel-eluting stent; SES, sirolimus-eluting stent; MASCOT, The Multinational Abluminal Sirolimus Coated Bio-engineered Stent ;TLR, target lesion revascularization; TVR, target vessel revascularization; NSTEMI, non-ST elevation myocardial infarction; STEMI, ST elevation myocardial infarction;

Supplementary Table S4 Baseline characteristics of participants

Characteristics	TICO		STOPDAPT-2		TWILIGHT		SMART-CHOICE	
	DAPT 3Mo+ P2Y12	DAPT 12Mo	DAPT 1Mo+ P2Y12	DAPT 12Mo	DAPT 3Mo+ P2Y12	DAPT 12Mo	DAPT 3Mo+ P2Y12	DAPT 12Mo
No. of participants	1527	1529	1500	1509	3555	3564	1495	1498
Age, mean(SD), years	61 (11)	61 (11)	68.1 (10.9)	69.1 (10.4)	65.2±10.3	65.1±10.4	64.6 (10.7)	64.4 (10.7)
Male, n/ total (%)	1204(79)	1224(80)	1183 (78.9)	1154 (76.5)	2709(76.2)	2712(76.1)	1087 (72.7)	1111 (74.2)
BMI, mean(SD), kg/m^2	24.9 (3.2)	24.9 (3.3)	24.4 (3.5)	24.2 (3.5)	28.6±5.5	28.5±5.6	24.5 (3.1)	24.7 (3.2)
Cardiovascular risk factors, n/total (%)								
Hypertension	760 (50)	781 (51)	1105 (73.7)	1116 (74.0)	2580 (72.6)	2574 (72.2)	921 (61.6)	919 (61.3)
Diabetes	418 (27)	417 (27)	585 (39.0)	574 (38.0)	1319(37.1)	1301(36.5)	570 (38.2)	552 (36.8)
Dyslipidemia	924 (61)	922 (60)	1116 (74.4)	1128 (74.8)	2157 (60.7)	2146 (60.2)	673 (45.1)	679 (45.5)
Current smoker	555 (36)	587 (38)	399 (26.6)	311 (20.6)	726 (20.4)	822 (23.1)	424 (28.4)	367 (24.5)
History, n/ total (%)								
MI	64 (4)	49 (3)	207 (13.8)	199 (13.2)	1020(28.7)	1020(28.6)	62 (4.1)	65 (4.3)
PCI	135 (9)	127 (8)	503 (33.5)	529 (35.1)	1502 (42.3)	1496 (42.0)	172 (11.5)	177 (11.8)
Coronary artery bypass graft	8 (1)	10 (1)	17 (1.1)	42 (2.8)	362 (10.2)	348 (9.8)	NA	NA
Stroke	60 (4)	66 (4)	81 (5.4)	105 (7.0)	NA	NA	99 (6.6)	102 (6.8)
Clinical presenta-tion, n/ total (%)								
Stable angina	NA	NA	935 (62.3)	926 (61.4)	1047 (29.5)	999 (28.0)	625 (41.8)	625 (41.8)
UA	442 (29)	484 (32)	193 (12.9)	214 (14.2)	1249 (35.1)	1245(34.9)	467 (31.2)	491 (32.8)
NSTEMA	539 (35)	488 (32)	81 (5.4)	99 (6.6)	1024 (28.8)	1096 (30.8)	239 (16.0)	230 (15.4)
STEMA	546 (36)	557 (36)	291 (19.4)	270 (17.9)	NA	NA	164 (11.0)	150 (10.0)
Target lesion site, n (%)								
Left main	49 (3)	45 (2)	43 (2.9)	37 (2.5)	166 (4.7)	187 (5.2)	23 (1.2)	35 (1.9)
Left anterior des-cending artery	912 (48)	909 (48)	828 (55.2)	854 (56.6)	1993 (56.1)	2010 (56.4)	903 (48.8)	950 (50.4)
Left circumflex artery	358 (19)	353 (19)	268 (17.9)	305 (20.2)	1151 (32.4)	1146 (32.2)	399 (21.6)	376 (19.9)
Right coronary artery	565 (30)	588 (31)	436 (29.1)	410 (27.2)	1243 (35.0)	1257 (35.3)	524 (28.3)	524 (27.8)

Characteristics	REDUCE		GLOBAL LEADERS		OPTIMA-C		DAPT-STEMI	
	DAPT 3Mo+ ASA	DAPT 12Mo	DAPT 1Mo+ P2Y12	DAPT 12Mo	DAPT 6Mo	DAPT 12Mo	DAPT 6Mo	DAPT 12Mo
No. of participants	751	745	7980	7988	683	684	433	437
Age, mean(SD), years	61.0	60.0	64.5(10.3)	64.6(10.3)	62.8±10.8	64.4±10.3	59.8±10.7	60.2±10.3
Male, n/ total (%)	620(82.6)	576(77.3)	6115 (76.6)	6139(76.9)	478 (70.0)	464 (67.8)	337 (78)	332 (76)
BMI, mean(SD), kg/m^2	26.6	26.6	28.2 (4.6)	28.2 (4.6)	NA	NA	27.8±4.3	27.9±4.5
Cardiovascular risk factors, n/total (%)								
Hypertension	379(50.7)	375(50.7)	5882(74.0)	5833(73.3)	426 (62.4)	437 (63.9)	193 (45)	195(45)
Diabetes	162(21.6)	145(19.5)	2049(25.7)	1989(24.9)	199 (29.1)	203 (29.7)	54 (13)	61(14)
Dyslipidemia	346(46.3)	333(44.9)	5345(69.3)	5423(70.0)	204 (29.9)	195 (28.5)	120 (28)	125(29)
Current smoker	313(42.1)	314(42.7)	2066(25.9)	2103(26.3)	184 (26.9)	184 (26.9)	218(51)	205(47)
History, n/ total (%)								
MI	NA	NA	1831(23.0)	1879(23.6)	18 (2.6)	25 (3.7)	26 (6)	20(5)
PCI	88(11.7)	73(9.8)	2609 (32.7)	2612(32.7)	59 (8.6)	71 (10.4)	29 (7)	18(4)
Coronary artery bypass graft	21(2.8)	21(2.8)	448(5.6)	495(6.2)	NA	NA	8 (2)	2(0.5)
Stroke	11(1.5)	15(2.0)	210(2.6)	211(2.6)	NA	NA	14 (3)	8(2)
Clinical presentation, n/ total (%)								
Stable angina	NA	NA	4230(53.0)	4251(53.2)	339 (49.6)	336 (49.1)	NA	NA
UA	114(15.2)	103(13.8)	1004(12.6)	1018(12.7)	254 (37.2)	253 (37.0)	NA	NA
NSTEMA	267(35.6)	305(41.0)	1684(21.1)	1689(21.1)	90 (13.2)	95 (13.9)	NA	NA
STEMA	370 (49.3)	336(45.2)	1062(13.3)	1030(12.9)	NA	NA	NA	NA
Target lesion site, n (%)								
Left main	NA	NA	197(1.9)	190 (1.8)	NA	NA	NA	NA
Left anterior descending artery	360(48)	329(44.2)	4283(41.2)	4383 (42.0)	394(57.7)	353(51.6)	169 (39)	188(43)
Left circumflex artery	146 (19.5)	164(22.0)	2524(24.3)	2553 (24.5)	134(19.6)	165(24.1)	89 (21)	70(16)
Right coronary artery	234(31.2)	246(33.0)	3284 (31.6)	3206 (30.7)	155(22.7)	166(24.3)	175 (41)	179(41)

Characteristics	SMART-DATE		NIPPON		IVUS-XPL		OPTIDUAL	
	DAPT 6Mo	L-DAPT	L-DAPT	DAPT 6Mo	DAPT 6Mo	DAPT 12Mo	L-DAPT	DAPT 12Mo
No. of participants	1357	1355	1653	1654	699	701	695	690
Age, mean(SD), years	62(54-71)	63(53-71)	67.2±9.9	67.4±9.6	63±9	64±9	64.1±10.8	64.2 ±11.5
Male, n/ total (%)	1016(74.9)	1028 (75.9)	1312(79.4)	1,304(78.8)	470(67)	494(70)	568(81.7)	547(79.3)
BMI, mean(SD), kg/m^2	24.3(3.2)	24.5(3.1)	24.3±3.5	24.5±3.5	24.8±3.1	24.6±3.0	NA	NA
Cardiovascular risk factors, n/total (%)								
Hypertension	669(49.9)	654(48.7)	1209 (73.1)	1177(71.2)	443(63)	455(65)	396(57.0)	417(60.4)
Diabetes	365(26.9)	379(28.1)	635(38.4)	619(37.4)	249(36)	257(37)	213(30.6)	222(32.2)
Dyslipidemia	322(24.2)	336(25.2)	1132(68.5)	1130(68.3)	473(68)	456(65)	NA	NA
Current smoker	506(38.0)	536(40.1)	997(60.3)	960(58.0)	171(25)	165(24)	425(61.2)	399(57.8)
History, n/ total (%)								
MI	30(2.3)	23(1.7)	195(11.8)	201(12.2)	34(5)	29(4)	119(17.1)	122(17.7)
PCI	65(4.9)	73(9.8)	432(26.1)	413(25.0)	72(10)	73(10)	180(25.9)	186(27.0)
Coronary artery bypass graft	NA	NA	29(1.8)	22(1.3)	22(3)	14(2)	37(5.3)	35(5.1)
Stroke	52(3.9)	58(4.4)	41(2.5)	48(2.9)	NA	NA	29(4.2)	25(3.6)
Clinical presentation, n/ total (%)								
Stable angina	NA	NA	734(44.4)	805(48.7)	356(51)	358(51)	240(34.5)	207(30.0)
UA	420 (31.0)	416(30.7)	330(20.0)	296(17.9)	237(34)	231(31)	66(9.5)	63(9.1)
NSTEMA	428 (31.5)	425(31.4)	26(1.6)	33(2.0)	NA	NA	99(14.2)	117(17.0)
STEMA	509 (37.5)	514(37.9)	196(11.9)	198(12.0)	NA	NA	74(10.7)	82(11.9)
Target lesion site, n (%)								
Left main	29(2.1)	17(1.3)	16(0.8)	7(0.4)	NA	NA	4(<1)	2(<1)
Left anterior descending artery	767(56.6)	826(61.0)	998(52.4)	981(51.8)	518(55)	530(56)	397(57)	443(64)
Left circumflex artery	331(24.4)	340(25.1)	374(19.7)	381(20.1)	187(20)	175(18)	225(32)	214(31)
Right coronary artery	504(37.2)	490(36.2)	515(27.1)	524(27.7)	239(25)	245(26)	280 (40)	268(39)

Characteristics	I-LOVE-IT 2		ISAR-SAFE		ITALIC		DAPT Study	
	**DAPT 6Mo**	**DAPT 12Mo**	**DAPT 6Mo**	**DAPT 12Mo**	**DAPT 6Mo**	**L-DAPT**	**L-DAPT**	**DAPT 12Mo**
No. of participants	909	920	1997	2003	926	924	5020	4941
Age, mean(SD), years	60.4±10.2	60.0±10.0	67.2(59.3–73.3)	67.2(59.1–73.7)	61.6±10.9	61.5±11.2	61.8±10.2	61.6±10.1
Male, n/ total (%)	611(67.2)	632(68.7)	1611(80.7)	1612(80.5)	750(81.0)	733(79.3)	3778(75.3)	3657(74.0)
BMI, mean(SD), kg/m^2	25.1±3.1	25.1±3.1	27.2(24.9–30.1)	27.5(24.9–30.4)	27.0±4.6	227.1±4.7	30.5±5.8	30.6±5.8
Cardiovascular risk factors, n/total (%)								
Hypertension	554(61.0)	596(64.8)	1797(90.1)	1830(91.5)	603(65.1)	594(64.3)	3796(75.8)	3649(74.0)
Diabetes	211(23.2)	203(22.1)	495(24.8)	484(24.2)	336(36.3)	349(37.8)	1556(31.1)	1481(30.1)
Dyslipidemia	230(25.3)	215(23.4)	1747(87.5)	1748(87.4)	625(67.5)	618(66.9)	NA	NA
Current smoker	333(36.6)	5352(38.3)	292(14.6)	306(15.3)	473(51.1)	487(52.7)	1222(24.62)	1210(24.7)
History, n/ total (%)								
MI	156(17.2)	145(15.8)	516(25.9)	491(24.5)	144(15.6)	138(14.9)	1092(22.0)	1026(21.1)
PCI	77(8.5)	60(6.5)	NA	NA	226(24.4)	209(22.6)	1518(30.4)	1529(31.0)
Coronary artery bypass graft	4(0.4)	4(0.4)	152(7.7)	149(7.5)	61(6.6)	45(4.9)	568(11.3)	581(11.8)
Stroke	84(9.2)	87(9.5)	NA	NA	28(3.0)	26(2.8)	155(3.1)	169(3.4)
Clinical presentation, n/ total (%)								
Stable angina	130(14.3)	139(15.1)	969(48.6)	956(47.8)	382(41.3)	383(41.5)	1882(37.5)	1870(37.8)
UA	527(58.0)	520(56.5)	429(21.5)	438(21.9)	188(20.3)	186(20.1)	838(16.7)	825(16.7)
NSTEMA	103(11.3)	98(10.7)	207(10.4)	203(10.1)	145(15.7)	152(16.5)	776(15.5)	767(15.5)
STEMA	122(13.4)	126(13.7)	158(7.9)	166(8.3)	67(7.2)	68(7.4)	534(10.6)	511(10.3)
Target lesion site, n (%)								
Left main	23(1.9)	21(1.7)	9(0.5)	3(0.2)	14(1.5)	8(0.9)	55(0.8)	55(0.9)
Left anterior descending artery	569(45.9)	569(45.3)	794(39.8)	812(40.6)	669(73.4)	658(72.3)	2715(41.2)	2586(40.4)
Left circumflex artery	284(22.9)	279(22.2)	528(26.4)	480(24.0)	456(50.0)	436(47.9)	1473(22.4)	1506(23.5)
Right coronary artery	364(29.4)	386(30.8)	636(31.8)	682(34.0)	489(53.6)	474(52.1)	2153(32.7)	2057(32.1)

Characteristics	DES LATE		SECURITY		ARCTIC-Interruption		OPTIMIZE	
	DAPT 12Mo	L-DAPT	DAPT 6Mo	DAPT 12Mo	L-DAPT	DAPT 12Mo	DAPT 3Mo+ ASA	DAPT 12Mo
No. of participants	2514	2531	682	717	636	624	1563	1556
Age, mean(SD), years	62.3±10.1	62.5±10.0	64.9±10.2	65.5±10.1	64(57–73)	64(57–73)	61.3(10.4)	61.9(10.6)
Male, n/ total (%)	1749(69.6)	1749(69.1)	529(77.6)	551(76.8)	508(80)	503(81)	992(63.5)	982(63.1)
BMI, mean(SD), kg/m^2	NA	NA	NA	NA	27(25-29)	27(25-30)	NA	NA
Cardiovascular risk factors, n/total (%)								
Hypertension	1423(56.6)	1479(58.4)	508(74.5)	510(71.1)	376(59)	388(62)	1350(86.4)	1371(88.2)
Diabetes	709(28.2)	709(28.0)	206(30.4)	223(31.7)	222(36)	349(37.8)	554(35.4)	549(35.3)
Dyslipidemia	NA	NA	446(65.4)	436(60.8)	428(67)	426(68)	953(63.2)	952(63.7)
Current smoker	722(28.7)	693(27.4)	139(20.5)	172(24.4)	147(23)	152(24)	290(18.6)	269(17.3)
History, n/ total (%)								
MI	92(3.7)	103(4.1)	145(21.2)	144(20.1)	197(31)	186(30)	541(34.6)	542(34.8)
PCI	276(11.0)	313(12.4)	132(19.4)	116(16.2)	273(43)	249(40)	327(20.9)	297(19.1)
Coronary artery bypass graft	NA	NA	38(5.6)	39(5.4)	47(7)	35(6)	111(7.1)	128(8.2)
Stroke	89(3.5)	15(4.5)	NA	NA	28(4)	38(6)	NA	NA
Clinical presenta-tion, n/ total (%)								
Stable angina	956(38.0)	1011(39.9)	341(61.6)	368(61.6)	NA	NA	935(59.8)	911(58.6)
UA	971(38.6)	930(36.7)	213(38.4)	229(38.4)	NA	NA	NA	NA
NSTEMA	266(10.6)	268(10.6)	NA	NA	NA	NA	84(5.4)	84(5.4)
STEMA	314(12.5)	314(12.4)	NA	NA	NA	NA	NA	NA
Target lesion site, n (%)								
Left main	90(2.6)	112(3.1)	NA	NA	18(3)	23(4)	24(1.2)	30(1.5)
Left anterior des-cending artery	1768(50.6)	1781(49.5)	402(43)	423(44)	342(54)	325(52)	986(47.9)	960(46.6)
Left circumflex artery	651(18.6)	715(19.9)	133(14.3)	137(14.2)	209(33)	181(29)	481(23.4)	501(24.3)
Right coronary artery	972(27.8)	976(27.1)	206(22)	207(21.6)	191(30)	222(36)	567(27.6)	571(27.7)

Characteristics	PRODIGY		RESET		EXCELLENT		REAL-ZEST LATE	
	L-DAPT	DAPT 6Mo	DAPT 3Mo+ASA	DAPT 12Mo	DAPT 6Mo	DAPT 12Mo	L-DAPT	DAPT 12Mo
No. of participants	987	983	1059	1058	722	721	1357	1344
Age, mean(SD), years	67.8±11	67.9±11	62.4±9.4	62.4±9.8	63.0±9.6	62.4±10.4	62.0±9.8	61.9±9.9
Male, n/ total (%)	764(77.4)	747(76.0)	682(64.4)	665(62.9)	470(65.1)	461(63.9)	950(70.0)	933(69.4)
BMI, mean(SD), kg/m^2	26.6	26.7	25.0±3.2	24.9±3.1	24.9±3.1	25.1±3.0	NA	NA
Cardiovascular risk factors, n/total (%)								
Hypertension	721(73.0)	693(70.4)	660(62.3)	650(61.4)	525(72.7)	532(73.8)	775(57.1)	765(56.9)
Diabetes	244(24.7)	233(23.7)	316(29.8)	305(28.8)	272(37.7)	278(38.6)	340(25.1)	364(27.1)
Dyslipidemia	553(56.0)	525(53.4)	611(57.7)	634(59.9)	543(75.2)	550(76.3)	586(43.2)	584(43.5)
Current smoker	222(22.5)	247(25.1)	267(25.2)	241(22.8)	198(27.4)	186(25.8)	404(29.8)	431(32.1)
History, n/ total (%)								
MI	270(27.3)	258(26.2)	19(1.8)	17(1.6)	47(6.5)	27(3.7)	51(3.8)	45(3.3)
PCI	184(18.6)	174(17.7)	37(3.5)	32(3.0)	67(9.3)	62(8.6)	177(13.0)	159(11.8)
Coronary artery bypass graft	110(11.1)	105(10.7)	2(0.2)	6(0.6)	11(1.5)	7(1.0)	NA	NA
Stroke	37(3.7)	39(4.0)	NA	NA	NA	NA	57(4.2)	45(3.3)
Clinical presenta-tion, n/ total (%)								
Stable angina	257(26.0)	250(25.4)	471(44.5)	490(46.3)	353(48.9)	346(48.0)	514(37.9)	514(37.9)
UA	183(18.5)	182(18.5)	432(40.8)	422(39.9)	NA	NA	514(37.9)	514(37.9)
NSTEMA	226(22.9)	224(22.8)	NA	NA	NA	NA	514(37.9)	144(10.7)
STEMA	321(32.5)	327(33.3)	NA	NA	19(2.6)	26(3.6)	155(11.4)	141(10.5)
Target lesion site, n (%)								
Left main	55(5.6)	56(5.7)	NA	NA	NA	NA	55(2.9)	44(2.4)
Left anterior descending artery	518(52.5)	518 (52.7)	707(52.7)	722(53.6)	452(63.0)	447(62.2)	912(48.7)	921(49.9)
Left circumflex artery	321(32.5)	318(32.4)	281(21.0)	259(19.2)	NA	NA	921(49.9)	334(18.1)
Right coronary artery	346(35.1)	363(36.9)	353(26.3)	365(27.1)	NA	NA	334(18.1)	546(29.6)

NA, the concrete information was not available; PCI, percutaneous coronary intervention; MI, myocardial infarction; ST, stent thrombosis; DAPT, dual antiplatelet therapy; L-DAPT, longer than 12 months DAPT; DAPT 12Mo,12 months DAPT; S-DAPT+ASA, shorter than 6-month DAPT followed by aspirin monotherapy; S-DAPT+P2Y12, shorter than 6-month DAPT followed by a P2Y12 receptor inhibitor monotherapy; BMI, body mass index (calculated as the weight in kilograms divided by height in meters); NSTEMI, non-ST elevation myocardial infarction; STEMI, ST elevation myocardial infarction; UA, unstable angina; SD, standard deviation

Supplementary Table S5 Definitions of clinical endpoints of the included trials

Trials	Cardiac death	MI	Definite or probable ST	Major Bleeding	Any Bleeding	NACE
**TICO**	Death due to MI, cardiac perforation or pericardial tamponade, an arrhythmia or conduction abnormality, stroke within 30 days of the procedure or related to the procedure, death due to a procedural.	Symptoms, electrocardiographic changes, or abnormal imaging findings, combined with a CK-MB fraction above the upper normal limits or a troponin T or troponin I level greater than the 99th percentile of the upper normal limit.	ARC criteria	TIMI major bleeding	TIMI minor or major	The composite of major bleeding and major adverse cardiac and cerebrovascular events.
**STOPDAPT-2**	All deaths are considered cardiovascular deaths unless a definite non-cardiovascular cause can be established.	ARC criteria	ARC criteria	TIMI major bleeding	TIMI major or minor bleeding	A composite of cardiovascular and bleeding events (cardiovascular death, MI, definite ST, ischemic or hemorrhagic stroke, or TIMI major or minor bleeding).
**TWILIGHT**	Any death due to proximate cardiac cause, unwitnessed death and death of unknown cause, all procedure-related deaths including those related to concomitant treatment.	Third universal definition	ARC criteria	BARC criteria type 3 or 5	BARC criteria type 2, 3 or 5	NA

**SMART-CHOICE**	All deaths were considered cardiac unless a definite non-cardiac cause could be established.	Elevated cardiac enzyme levels above the upper reference limit with ischemic symptoms or electrocardiographic findings indicative of ischemia.	ARC criteria	BARC criteria type 3,4 or 5 bleeding	BARC criteria type 2 to 5 bleeding	Major adverse cardiac and cerebrovascular events plus BARC type 2 to 5 bleeding.
**REDUCE**	All deaths are considered cardiovascular deaths unless a definite non-cardiovascular cause can be established.	Third universal definition of MI	ARC Criteria	NA	BARC criteria type 2, 3 or 5	Composite of all-cause mortality, MI, ST, stroke, TVR or bleeding (BARC 2, 3, 5).
**GLOBAL LEADERS**	All deaths were considered cardiac unless a definite non-cardiac cause could be established.	Third universal MI definition	ARC criteria	BARC criteria type 3 or 5	BARC criteria type 2, 3 or 5	A composite of all-cause mortality, any stroke, MI, revascularization, BARC type 3 or 5 bleeding.
**OPTIMA-C**	ARC criteria	ARC criteria	ARC criteria	TIMI major bleeding	NA	NA
**DAPT-STEMI**	All deaths are considered cardiac unless an unequivocal non-cardiac cause can be established.	ARC criteria	ARC criteria	TIMI major bleeding	TIMI criteria	All-cause mortality, any MI, any revascularization, stroke, and TIMI major bleeding.

**SMART-DATE**	All deaths were considered cardiac unless a definite non-cardiac cause could be established.	Elevated cardiac enzymes above the upper reference limit with ischemic symptoms or electrocardiography findings indicative of ischemia that was not related to the index procedure.	ARC criteria	BARC type 3-5 bleeding	BARC type 2-5 bleeding	Major adverse cardiac and cerebrovascular events plus BARC type 2–5 bleeding.
**NIPPON**	All deaths are considered to be cardiac deaths unless an unequivocal non-cardiac cause can be established.	(1)Per-procedural: A serum CK-MB level exceeding the ULN should not be considered as new MI, but as MI at registration; a serum troponin or serum CK-MB level exceeding 3 times the ULN within 48 hours after PCI; a serum troponin or serum CK-MB level exceeding 5 times the ULN within 72 hours after CABG; and a new Q-wave, left bundle block, new occlusion of the native vessel or graft, or reduction of viable myocardium on diagnostic imaging.(2)Spontaneous: When myocardial enzymes are at or above the ULN, it should be considered as MI at registration, and when the serum level of troponin or CK-MB exceeds ULN more than 48 hours after PCI or within 72 hours after CABG.(3)Re-infarction: Blood levels of biomarkers measured twice after the onset of MI are stable or decrease and the values at 3 to 6 hours after PCI show a > 20% increase compared with those obtained at index PCI.	ARC criteria	BARC 3 and 5 type bleeding	NA	Composite event of all-cause death, nonfatal MI, nonfatal stroke, or major bleeding.
**ITALIC**	All deaths unless an unequivocal non-cardiac cause could be established.	Q-wave MI was defined by recurrence of symptoms and/or development of new pathological Q waves in 2	ARC criteria	TIMI major bleeding	TIMI Major or Minor	A composite of all-cause mortality, MI, stroke, TVR,

		or more contiguous leads with elevated CK, CK-MB, or troponin levels. Non–Q-wave MI was defined by >2-fold CK elevation with elevated CK-MB or troponin without new pathological Q waves.			Bleeding	major bleeding.
**IVUS-XPL**	All deaths were considered cardiac deaths unless a definite noncardiac cause could be established.	The presence of consistent clinical symptoms, electrocardiographic changes, or abnormal imaging findings, combined with a CK-MB fraction increase greater than the upper normal limit or an increase in troponin T or troponin I to >99th percentile of the upper normal limit.	ARC criteria	TIMI major bleeding	NA	Composite of cardiac death, MI, stroke, or TIMI major bleeding.
**I-LOVE-IT 2**	Any death due to an evident cardiac cause, any death related to PCI, unwitnessed death or death of unknown causes.	Typical rise and fall of troponin or CK-MB fraction with at least one of the following: ischemic symptoms, development of pathological Q waves, ischemic electrocardiographic changes, or pathological findings of an acute MI.	ARC criteria	BARC type≥3 bleeding	BARC criteria	A composite of all-cause death, all MI, stroke, and major bleeding (BARC type ≥3 bleeding).
**OPTIDUAL**	NA	The presence of clinical or electrocardiographic changes consistent with myocardial ischemia in the setting of increased cardiac biomarkers above the upper incorporates confounders to account for potential imbalances due to limit of normal in accordance with the universal definition.	ARC criteria	TIMI major bleeding	TIMI criteria	Composite of death, MI, stroke, and major bleeding.
**ISAR-SAFE**	NA	TIMI Criteria	ARC criteria	BARC criteria type 3 or 5	BARC criteria type 2, 3 or 5	The composite of death, MI, definite, or probable ST, stroke or TIMI major bleeding.

**DAPT Study**	Any death due to immediate cardiac cause. Unwitnessed death and death of unknown cause will be classified as cardiac death.	The categories include per-procedual PCI, per-procedual CABG, spontaneous, silent, sudden death, and reinfarction, more details were provided in the online appendix of the original article.	ARC criteria	GUSTO severe bleeding	BARC criteria	NA
**DES LATE**	All deaths were considered to have resulted from cardiac causes unless an unequivocal non-cardiac cause could be established.	Universal definition of myocardial infarction	ARC criteria	TIMI major bleeding	NA	A composite of cardiac death, MI, stroke, ST, or TIMI major bleeding.
**SECURITY**	Any death without a non-cardiac cause.	Cardiac enzyme elevation above the upper normal limit associated with at least one ischemic symptom; development of Q waves on the electrocardiogram; electrocardiogram changes indicative of ischemia or coronary artery intervention	ARC criteria	BARC 3 or 5 type bleeding	BARC criteria	A composite of cardiac death, MI, stroke, definite or probable ST, or BARC type 2,3 or 5 bleeding.
**ARCTIC-Interruption**	NA	In patients with elevated biomarkers before PCI, positive diagnosis of reinfarction is made when all of the following criteria are present: (1) documentation that troponin level was decreasing; (2) troponin measured 6 hours after PCI is N3×ULN; (3) peak troponin level measured within 24 hours after the event is elevated by at least 50% above the previous level. In patients in whom biomarkers are normal or have returned to normal prior to PCI, periprocedural MI is defined when troponin measured 6 hours after PCI is N3×ULN. Measurements of biomarkers are requested before and 6 hours after PCI and at discharge.	ARC criteria	STEEPLE major bleeding	STEEPLE criteria	Any death, MI, ST, stroke or TIA, urgent revascularisation, TIMI major bleeding.

**OPTIMIZE**	Any unknown causes of death or death that cannot be clearly attributed to a non-cardiac cause will be considered cardiac.	Classified as Q wave or non-Q wave; Periprocedural—within 48 hours post-PCI with baseline biomarker <ULN, rise in CKMB or troponin >3 times ULN; Spontaneous—CK-MB or troponin >ULN; Re-infarction—stable or decreasing biomarker values on 2 samples and >20% increase 3 to 6 hours post-intervention as compared to baseline samples.	ARC criteria	Intracranial, intraocular, or retroperitoneal hemorrhage; Clinically overt blood loss resulting in a decree-se in hemoglobin of more than 3g/d; Any decrease in hemoglobin of more than 4 g/dL; Transfusion of 1 or more units of packed red blood cells or whole blood; Bleeding causes hemodynamic compromise and requires intervention.	Major bleeding plus bleeding events that did not meet criteria for either major or severe or life-threatening bleeding.	Composite of death from all causes, MI, stroke, or major bleeding.
**PRODIGY**	All deaths were considered to be of cardiovascular causes unless an unequivocal noncardiovascular cause	Universal definition of myocardial infarction	ARC criteria	BARC type 3-5 bleeding	BARC type 2-5 bleeding	NA

	could be established.					
**RESET**	All deaths are considered cardiovascular deaths unless a definite non-cardiovascular cause can be established.	Presence of clinical symptoms, electrocardiographic change or abnormal imaging findings of myocardial infarction combined with an increase in creatine kinase myocardial band fraction to greater than three times the upper limit of the normal range or troponin-T/troponin-I more than the 99th percentile of the upper normal limit, unrelated to an interventional procedure.	ARC criteria	TIMI major bleeding	TIMI criteria	A composite of cardiovascular death, MI, ST, TVR, or bleeding.
**EXCELLENT**	All deaths were considered cardiac unless a definite non-cardiac cause could be established.	During the first 48 hours after PCI, defined as an increase of cardiac enzyme 3 times above the upper limit of normal in stable patients; In patients with elevated baseline levels of cardiac enzyme, myocardial infarction was defined as a subsequent increase of >2-fold from baseline values; After the first 48hours, myocardial infarction was defined as the presence of clinical signs of MI combined with a CK-MB fraction or troponin T/troponin I increase higher than upper limit of normal.	ARC criteria	TIMI major bleeding	TIMI criteria	A composite of death, MI,stroke, ST, or TIMI major bleeding.
**REAL-ZEST LATE**	All deaths were considered cardiac unless a definite non-cardiac cause could be established.	Universal definition of myocardial infarction	ARC criteria	TIMI major bleeding	NA	NA

NACE, net adverse clinical events; NA, the concrete definition was not available; MI, myocardial infarction; ARC, Academic Research Consortium; TIMI, thrombolysis in myocardial Infarction; BARC, Bleeding Academic Research Consortium; CK-MB, creatine kinase-myocardial band; GUSTO, The Global Use of Strategies to Open Occluded Arteries; STEEPLE, Enoxaparin versus Unfractionated Heparin in Elective Percutaneous Coronary Intervention; REPLACE, Randomized Evaluation of PCI Linking Angiomax to Reduced Clinical Events; ISTH, International Society on Thrombosis and Haemostasi

Supplementary Table S6 The number of participants who had each outcome in each study

Trial	Treatment	Total	All-cause mortality	Cardiac death	MI	Major bleeding	Any bleeding	Definite or probable ST	NACE
TICO	DAPT 3Mo+P2Y12	1527	16	7	6	25	53	6	59
	DAPT 12Mo	1529	23	12	11	45	83	4	89
STOPDAPT-2	DAPT 1Mo+P2Y12	1500	21	9	13	3	6	4	35
	DAPT 12Mo	1509	18	11	11	16	23	1	55
TWILIGHT	DAPT 3Mo+P2Y12	3555	34	26	95	34	141	14	NA
	DAPT 12Mo	3564	45	37	95	69	250	19	NA
SMART-CHOICE	DAPT 3Mo+P2Y12	1495	21	11	11	12	28	3	65
	DAPT 12Mo	1498	18	13	17	14	49	2	81
REDUCE	DAPT 3Mo+ASA	733	23	13	26	24	24	12	85
	DAPT 12Mo	727	16	8	22	29	29	6	88
GLOBAL LEADERS	DAPT 1Mo+P2Y12	7980	224	NA	248	163	529	64	616
	DAPT 12Mo	7988	253	NA	250	169	532	64	653
OPTIMA-C	DAPT 6Mo	683	2	1	1	1	1	0	NA
	DAPT 12Mo	684	3	2	1	1	1	1	NA
DAPT-STEMI	DAPT 6Mo	433	3	2	8	1	3	3	21
	DAPT 12Mo	437	6	4	8	2	5	4	29
SMART-DATE	DAPT 6Mo	1357	35	18	24	6	35	15	96
	L-DAPT	1355	39	24	10	10	51	10	99

NIPPON	L-DAPT	1653	7	4	1	12	12	1	24
	DAPT 6Mo	1654	16	8	4	11	11	2	34
IVUS-XPL	DAPT 6Mo	699	5	3	1	5	5	2	15
	DAPT 12Mo	701	10	5	0	7	7	2	14
OPTIDUAL	L-DAPT	695	16	10	11	4	18	3	40
	DAPT 12Mo	690	24	14	16	4	20	1	52
I-LOVE-IT 2	DAPT 6Mo	909	14	7	42	13	57	5	72
	DAPT 12Mo	920	17	8	37	7	60	2	67
ISAR-SAFE	DAPT 6Mo	1997	8	NA	13	6	21	5	29
	DAPT 12Mo	2003	12	NA	14	22	46	4	32
ITALIC	DAPT 6Mo	926	11	5	12	0	6	6	32
	L-DAPT	924	20	5	9	4	10	3	34
DAPT Study	L-DAPT	5020	98	45	99	119	263	19	NA
	DAPT 12Mo	4941	74	47	198	73	137	65	NA
DES LATE	DAPT 12Mo	2514	32	19	27	24	24	11	74
	L-DAPT	2531	46	28	19	34	34	7	89
SECURITY	DAPT 6Mo	682	14	6	21	5	6	3	46
	DAPT 12Mo	717	15	6	19	8	10	3	45
ARCTIC-Interruption	L-DAPT	635	7	NA	9	7	12	0	30
	DAPT 12Mo	624	9	NA	9	1	3	3	28
OPTIMIZE	DAPT 3Mo+ASA	1563	43	29	49	10	35	13	93
	DAPT 12Mo	1556	45	32	42	14	45	12	90
PRODIGY	L-DAPT	987	65	36	39	34	73	13	NA
	DAPT 6Mo	983	65	37	41	21	35	15	NA
RESET	DAPT 3Mo+ASA	1059	5	2	2	2	5	2	40

	DAPT 12Mo	1058	8	4	4	6	10	3	41
EXCELLENT	DAPT 6Mo	722	4	2	13	2	4	6	24
	DAPT 12Mo	721	7	3	7	4	10	1	21
REAL-ZEST LATE	L-DAPT	1357	20	13	10	3	3	5	NA
	DAPT 12Mo	1344	13	8	7	1	1	4	NA

NA, the concrete outcomes was not available; MI, myocardial infarction; ST, stent thrombosis; ANCE, net adverse clinical events; DAPT, dual antiplatelet therapy; L-DAPT, longer than 12 months DAPT; DAPT 12Mo,12 months DAPT; DAPT 6Mo,6 months DAPT; DAPT 3Mo+ASA, 3-months DAPT followed by aspirin monotherapy; DAPT 3Mo+P2Y12, 3-month DAPT followed by a P2Y12 receptor inhibitor monotherapy; DAPT 1Mo+P2Y12,1-month DAPT followed by a P2Y12 receptor inhibitor monotherapy.

Supplementary Table S7 Risk of bias of included trials using the Cochrane Risk Assessment Tool

Trial	Random sequence generation (selection bias)	Allocation concealment (selection bias)	Blinding of participants and personnel* (performance bias)	Blinding of outcome assessment (detection bias)	Incomplete outcome data** (attrition bias)	Selective reporting (reporting bias)	Other sources of bias
TICO	+	+	?	+	+	+	?
STOPDAPT-2	+	?	?	+	+	+	?
TWILIGHT	+	+	+	+	+	+	?
SMART-CHOICE	+	+	?	+	+	+	?
REDUCE	+	+	-	?	+	+	-
GLOBAL LEADERS	+	+	+	?	+	+	?
OPTIMA-C	+	+	+	+	+	+	?
DAPT-STEMI	+	?	?	+	+	+	?
SMART-DATE	+	+	+	?	+	+	?
NIPPON	+	+	?	+	+	+	?
ITALIC	+	?	?	+	+	+	?
IVUS-XPL	+	+	?	+	+	+	-
I-LOVE-IT 2	+	?	?	?	+	+	?
OPTIDUAL	+	+	+	+	+	+	?
ISAR-SAFE	+	+	+	+	+	+	?
DAPT Study	?	?	+	-	+	+	?

DES LATE	+	+	-	-	+	+	-
SECURITY	+	+	?	?	?	+	-
ARCTIC-Interruption	+	+	+	?	+	+	?
OPTIMIZE	+	+	+	+	+	+	?
PRODIGY	+	+	?	?	+	+	?
RESET	+	+	?	?	+	+	-
EXCELLENT	+	+	?	?	+	+	-
REAL-ZEST LATE	+	+	-	-	+	+	-

*Despite that the trials used an open label trial design, the outcome assessment was blinded, and therefore the study design did not influence reported outcomes and hereby negatively effecting the quality of evidence.

**All trials reported minimal loss to follow-up.

Green refers to a low risk of bias; yellow refers to the uncertain risk of bias; red refers to a high risk of bias.

Supplementary Table S8 Pooled estimates of the network meta-analysis of each endpoint. Results in the upper triangle are odds ratios (95% confidence intervals) from the network meta-analysis between the column defining intervention and row defining intervention. Significant results are in bold.

All-cause mortality

DAPT 12Mo	0.93 (0.66, 1.40)	1.00 (0.66, 1.55)	0.84 (0.57, 1.25)	0.89 (0.64, 1.21)	1.06 (0.79, 1.35)
1.07 (0.72, 1.52)	DAPT 1Mo+P2Y12	1.08 (0.60, 1.88)	0.89 (0.51, 1.52)	0.96 (0.55, 1.48)	1.13 (0.67, 1.71)
1.00 (0.64, 1.51)	0.93 (0.53, 1.66)	DAPT 3Mo+ASA	0.84 (0.46, 1.46)	0.89 (0.50, 1.48)	1.06 (0.62, 1.70)
1.19 (0.80, 1.77)	1.12 (0.66, 1.96)	1.20 (0.68, 2.18)	DAPT 3Mo+P2Y12	1.07 (0.62, 1.70)	1.27 (0.76, 2.01)
1.12 (0.83, 1.57)	1.04 (0.68, 1.82)	1.12 (0.68, 1.99)	0.94 (0.59, 1.60)	DAPT 6Mo	1.18 (0.91, 1.59)
0.95 (0.74, 1.27)	0.88 (0.59, 1.49)	0.94 (0.59, 1.62)	0.79 (0.50, 1.31)	0.85 (0.63, 1.10)	L-DAPT

Cardiac death

DAPT 12Mo	0.78 (0.31, 1.99)	0.99 (0.61, 1.63)	0.70 (0.45, 1.09)	0.93 (0.63, 1.39)	1.02 (0.73, 1.40)
1.28 (0.50, 3.23)	DAPT 1Mo+P2Y12	1.26 (0.45, 3.55)	0.90 (0.32, 2.58)	1.19 (0.42, 3.26)	1.32 (0.48, 3.45)
1.01 (0.61, 1.64)	0.80 (0.28, 2.20)	DAPT 3Mo+ASA	0.73 (0.35, 1.37)	0.97 (0.49, 1.76)	1.04 (0.57, 1.87)
1.43 (0.92, 2.23)	1.12 (0.39, 3.09)	1.37 (0.73, 2.87)	DAPT 3Mo+P2Y12	1.31 (0.73, 2.37)	1.45 (0.84, 2.46)
1.08 (0.72, 1.59)	0.84 (0.31, 2.39)	1.03 (0.57, 2.03)	0.76 (0.42, 1.38)	DAPT 6Mo	1.11 (0.78, 1.54)
0.98 (0.71, 1.36)	0.76 (0.29, 2.10)	0.96 (0.53, 1.76)	0.69 (0.41, 1.19)	0.90 (0.65, 1.29)	L-DAPT

Myocardial infarction

DAPT 12Mo	1.03 (0.66, 1.70)	1.10 (0.69, 1.75)	0.84 (0.52, 1.27)	1.12 (0.82, 1.57)	**0.68 (0.51, 0.95)**
0.97 (0.59, 1.51)	DAPT 1Mo+P2Y12	1.07 (0.53, 2.04)	0.82 (0.40, 1.45)	1.08 (0.63, 1.92)	0.66 (0.38, 1.17)
0.91 (0.57, 1.46)	0.93 (0.49, 1.90)	DAPT 3Mo+ASA	0.76 (0.39, 1.41)	1.01 (0.59, 1.84)	0.62 (0.36, 1.12)
1.19 (0.79, 1.93)	1.22 (0.69, 2.51)	1.32 (0.71, 2.56)	DAPT 3Mo+P2Y12	1.33 (0.80, 2.45)	0.81 (0.50, 1.51)

0.89 (0.64, 1.22)	0.93 (0.52, 1.60)	0.99 (0.54, 1.71)	0.75 (0.41, 1.25)	DAPT 6Mo	**0.61 (0.43, 0.87)**
**1.47 (1.06, 1.95)**	1.52 (0.86, 2.62)	1.62 (0.89, 2.78)	1.24 (0.66, 2.02)	**1.63 (1.15, 2.34)**	L-DAPT
**Major bleeding**					
DAPT 12Mo	0.67 (0.26, 1.23)	0.67 (0.31, 1.31)	0.58 (0.32, 1.07)	0.77 (0.46, 1.23)	1.50 (0.95, 2.54)
1.49 (0.81, 3.92)	DAPT 1Mo+P2Y12	1.01 (0.39, 3.15)	0.86 (0.37, 2.81)	1.14 (0.52, 3.39)	**2.22 (1.07, 7.12)**
1.49 (0.76, 3.27)	0.99 (0.32, 2.59)	DAPT 3Mo+ASA	0.85 (0.36, 2.37)	1.15 (0.50, 2.86)	**2.26 (1.02, 5.82)**
1.74 (0.94, 3.11)	1.16 (0.36, 2.68)	1.18 (0.42, 2.77)	DAPT 3Mo+P2Y12	1.33 (0.60, 2.75)	**2.62 (1.21, 5.63)**
1.31 (0.81, 2.17)	0.87 (0.29, 1.91)	0.87 (0.35, 2.01)	0.75 (0.36, 1.67)	DAPT 6Mo	**1.98 (1.21, 3.50)**
0.67 (0.39, 1.05)	**0.45 (0.14, 0.93)**	**0.44 (0.17, 0.98)**	**0.38 (0.18, 0.82)**	**0.50 (0.29, 0.83)**	L-DAPT
**Any bleeding**					
DAPT 12Mo	0.74 (0.38, 1.18)	0.74 (0.43, 1.22)	**0.58 (0.38, 0.88)**	0.76 (0.52, 1.04)	**1.48 (1.02, 2.03)**
1.35 (0.85, 2.65)	DAPT 1Mo+P2Y12	1.00 (0.51, 2.29)	0.77 (0.43, 1.77)	1.02 (0.58, 2.11)	**1.99 (1.13, 4.14)**
1.35 (0.82, 2.30)	1.00 (0.44, 1.95)	DAPT 3Mo+ASA	0.79 (0.41, 1.54)	1.03 (0.56, 1.88)	**2.01 (1.07, 3.67)**
**1.73 (1.14, 2.63)**	1.29 (0.57, 2.32)	1.27 (0.65, 2.44)	DAPT 3Mo+P2Y12	1.31 (0.75, 2.20)	**2.56 (1.46, 4.29)**
1.32 (0.96, 1.91)	0.98 (0.47, 1.71)	0.97 (0.53, 1.80)	0.76 (0.45, 1.34)	DAPT 6Mo	**1.95 (1.37, 2.74)**
**0.68 (0.49, 0.98)**	**0.50 (0.24, 0.89)**	**0.50 (0.27, 0.94)**	**0.39 (0.23, 0.68)**	**0.51 (0.37, 0.73)**	L-DAPT
**Definite or probable stent thrombosis**					
DAPT 12Mo	1.20 (0.54, 3.65)	1.25 (0.54, 2.95)	1.00 (0.44, 2.46)	1.09 (0.62, 2.08)	0.58 (0.34, 1.11)
0.83 (0.27, 1.85)	DAPT 1Mo+P2Y12	1.03 (0.25, 3.28)	0.82 (0.21, 2.75)	0.89 (0.28, 2.53)	0.47 (0.15, 1.35)
0.80 (0.34, 1.87)	0.97 (0.30, 3.96)	DAPT 3Mo+ASA	0.79 (0.25, 2.80)	0.87 (0.32, 2.55)	0.46 (0.18, 1.38)
1.00 (0.41, 2.29)	1.23 (0.36, 4.73)	1.26 (0.36, 4.05)	DAPT 3Mo+P2Y12	1.10 (0.39, 3.10)	0.58 (0.21, 1.67)
0.92 (0.48, 1.62)	1.12 (0.39, 3.58)	1.15 (0.39, 3.13)	0.91 (0.32, 2.58)	DAPT 6Mo	**0.53 (0.29, 0.98)**
1.73 (0.90, 2.91)	2.11 (0.74, 6.59)	2.16 (0.72, 5.66)	1.72 (0.60, 4.73)	**1.88 (1.02, 3.40)**	L-DAPT
**Net adverse clinical events**					
DAPT 12Mo	0.89 (0.68, 1.06)	0.98 (0.78, 1.24)	**0.72 (0.53, 0.95)**	1.02 (0.84, 1.22)	1.00 (0.80, 1.23)

1.12 (0.94, 1.47)	DAPT 1Mo+P2Y12	1.11 (0.82, 1.60)	0.81 (0.58, 1.20)	1.15 (0.89, 1.59)	1.12 (0.86, 1.56)
1.02 (0.81, 1.29)	0.90 (0.63, 1.21)	DAPT 3Mo+ASA	0.73 (0.51, 1.05)	1.04 (0.77, 1.40)	1.02 (0.74, 1.38)
**1.40 (1.05, 1.87)**	1.23 (0.83, 1.74)	1.37 (0.95, 1.96)	DAPT 3Mo+P2Y12	**1.42 (1.01, 2.04)**	1.40 (0.97, 1.95)
0.98 (0.82, 1.19)	0.87 (0.63, 1.13)	0.96 (0.72, 1.31)	**0.70 (0.49, 0.99)**	DAPT 6Mo	0.98 (0.80, 1.20)
1.00 (0.82, 1.24)	0.89 (0.64, 1.17)	0.98 (0.72, 1.36)	0.72 (0.51, 1.03)	1.02 (0.83, 1.26)	L-DAPT

Supplementary Table S9 Node-splitting analysis of Inconsistency for all endpoints

Endpoints	Comparison	Direct effect	Indirect effect	Overall	P-value
All-cause mortality	DAPT 12Mo vs DAPT 6Mo	-0.33 (-0.74, 0.04)	0.13 (-0.30, 0.57)	-0.11 (-0.45, 0.19)	0.09
	DAPT 12Mo vs L-DAPT	0.17 (-0.14, 0.45)	-0.31 (-0.86, 0.20)	0.05 (-0.24, 0.30)	0.10
	DAPT 6Mo vs L-DAPT	0.02 (-0.28, 0.34)	0.51 (0.02, 0.99)	0.16 (-0.10, 0.46)	0.12
Cardiac death	DAPT 12Mo vs DAPT 6Mo	-0.29 (-0.91, 0.28)	0.09 (-0.46, 0.65)	-0.08 (-0.47, 0.33)	0.33
	DAPT 12Mo vs L-DAPT	0.08 (-0.27, 0.44)	-0.30 (-1.03, 0.42)	0.02 (-0.31, 0.34)	0.34
	DAPT 6Mo vs L-DAPT	0.00 (-0.40, 0.39)	0.37 (-0.29, 1.07)	0.10 (-0.25, 0.43)	0.32
Myocardial infarction	DAPT 12Mo vs DAPT 6Mo	0.18 (-0.18, 0.54)	-0.08 (-0.63, 0.57)	0.11 (-0.20, 0.45)	0.42
	DAPT 12Mo vs L-DAPT	-0.47 (-0.79, -0.05)	-0.19 (-0.79, 0.36)	-0.38 (-0.67, -0.06)	0.44
	DAPT 6Mo vs L-DAPT	-0.38 (-0.83, 0.04)	-0.65 (-1.10, -0.09)	-0.49 (-0.85, -0.14)	0.41
Major bleeding	DAPT 12Mo vs DAPT 6Mo	-0.41 (-1.00, 0.19)	0.02 (-0.86, 0.92)	-0.27 (-0.77, 0.21)	0.40
	DAPT 12Mo vs L-DAPT	0.52 (0.00, 1.16)	0.10 (-0.76, 1.03)	0.41 (-0.05, 0.93)	0.39
	DAPT 6Mo vs L-DAPT	0.53 (-0.10, 1.25)	0.95 (0.16, 1.85)	0.68 (0.19, 1.25)	0.40
Any bleeding	DAPT 12Mo vs DAPT 6Mo	-0.44 (-0.87, -0.05)	0.00 (-0.59, 0.63)	-0.28 (-0.65, 0.04)	0.20
	DAPT 12Mo vs L-DAPT	0.52 (0.11, 0.93)	0.07 (-0.57, 0.65)	0.39 (0.02, 0.71)	0.19
	DAPT 6Mo vs L-DAPT	0.51 (0.05, 0.94)	0.96 (0.40, 1.56)	0.67 (0.32, 1.01)	0.19
Definite or probable stent thrombosis	DAPT 12Mo vs DAPT 6Mo	0.36 (-0.37, 1.07)	-0.41 (-1.23, 0.68)	0.09 (-0.48, 0.73)	0.23
	DAPT 12Mo vs L-DAPT	-0.78 (-1.37, -0.04)	-0.02 (-1.04, 0.95)	-0.55 (-1.07, 0.11)	0.25
	DAPT 6Mo vs L-DAPT	-0.39 (-1.12, 0.29)	-1.12 (-2.07, -0.08)	-0.63 (-1.22, -0.02)	0.25
Net adverse clinical	DAPT 12Mo vs DAPT 6Mo	-0.00 (-0.22, 0.22)	0.04 (-0.32, 0.44)	0.02 (-0.17, 0.20)	0.81

events	DAPT 12Mo vs L-DAPT	0.02 (-0.26, 0.27)	-0.04 (-0.40, 0.32)	-0.00 (-0.22, 0.20)	0.80
	DAPT 6Mo vs L-DAPT	-0.03 (-0.32, 0.23)	0.01 (-0.36, 0.37)	-0.02 (-0.23, 0.18)	0.87

DAPT, dual antiplatelet therapy; L-DAPT, longer than 12 months DAPT; DAPT 12Mo,12 months DAPT; DAPT 6Mo,6 months DAPT; DAPT 3Mo+ASA, 3-months DAPT followed by aspirin monotherapy; DAPT 3Mo+P2Y12, 3-month DAPT followed by a P2Y12 receptor inhibitor monotherapy; DAPT 1Mo+P2Y12,1-month DAPT followed by a P2Y12 receptor inhibitor monotherapy.

Supplementary Table S10 Pooled estimates of the network meta-analysis of sensitivity analysis

All-cause mortality

DAPT 12Mo	0.93 (0.63, 1.49)	1.03 (0.65, 1.61)	0.82 (0.55, 1.26)	0.87 (0.61, 1.19)	1.05 (0.77, 1.37)
1.08 (0.67, 1.58)	DAPT 1Mo+P2Y12	1.11 (0.57, 1.97)	0.89 (0.48, 1.58)	0.93 (0.50, 1.51)	1.13 (0.63, 1.78)
0.97 (0.62, 1.54)	0.90 (0.51, 1.77)	DAPT 3Mo+ASA	0.80 (0.44, 1.50)	0.85 (0.47, 1.45)	1.02 (0.59, 1.71)
1.22 (0.79, 1.82)	1.13 (0.63, 2.10)	1.26 (0.67, 2.27)	DAPT 3Mo+P2Y12	1.05 (0.59, 1.73)	1.28 (0.74, 2.07)
1.15 (0.84, 1.64)	1.07 (0.66, 1.99)	1.18 (0.69, 2.11)	0.95 (0.58, 1.68)	DAPT 6Mo	1.21 (0.87, 1.69)
0.95 (0.73, 1.30)	0.88 (0.56, 1.60)	0.98 (0.58, 1.70)	0.78 (0.48, 1.34)	0.83 (0.59, 1.15)	L-DAPT

Cardiac death

DAPT 12Mo	0.78 (0.31, 2.13)	1.00 (0.60, 1.68)	0.72 (0.45, 1.10)	0.97 (0.63, 1.44)	1.00 (0.72, 1.39)
1.28 (0.47, 3.19)	DAPT 1Mo+P2Y12	1.26 (0.41, 3.71)	0.90 (0.29, 2.47)	1.23 (0.42, 3.41)	1.28 (0.45, 3.26)
1.00 (0.60, 1.65)	0.79 (0.27, 2.46)	DAPT 3Mo+ASA	0.73 (0.36, 1.40)	0.98 (0.51, 1.85)	1.00 (0.55, 1.80)
1.39 (0.91, 2.23)	1.12 (0.40, 3.40)	1.37 (0.71, 2.79)	DAPT 3Mo+P2Y12	1.33 (0.74, 2.49)	1.40 (0.81, 2.52)
1.03 (0.69, 1.58)	0.81 (0.29, 2.40)	1.02 (0.54, 1.96)	0.75 (0.40, 1.35)	DAPT 6Mo	1.03 (0.70, 1.53)
1.00 (0.72, 1.39)	0.78 (0.31, 2.22)	1.00 (0.56, 1.82)	0.71 (0.40, 1.24)	0.97 (0.65, 1.44)	L-DAPT

Myocardial infarction

DAPT 12Mo	1.03 (0.66, 1.63)	1.11 (0.69, 1.75)	0.84 (0.52, 1.27)	1.06 (0.78, 1.49)	**0.69 (0.52, 0.99)**
0.97 (0.61, 1.50)	DAPT 1Mo+P2Y12	1.08 (0.55, 2.01)	0.82 (0.42, 1.45)	1.02 (0.60, 1.83)	0.67 (0.40, 1.20)
0.90 (0.57, 1.46)	0.92 (0.50, 1.80)	DAPT 3Mo+ASA	0.77 (0.39, 1.43)	0.95 (0.56, 1.73)	0.62 (0.37, 1.14)
1.18 (0.79, 1.93)	1.22 (0.69, 2.38)	1.30 (0.70, 2.54)	DAPT 3Mo+P2Y12	1.25 (0.75, 2.34)	0.82 (0.49, 1.54)
0.95 (0.67, 1.28)	0.98 (0.55, 1.67)	1.05 (0.58, 1.79)	0.80 (0.43, 1.34)	DAPT 6Mo	**0.65 (0.46, 0.96)**
**1.45 (1.01, 1.93)**	1.50 (0.83, 2.48)	1.60 (0.88, 2.70)	1.22 (0.65, 2.05)	**1.53 (1.04, 2.17)**	L-DAPT

Major bleeding

DAPT 12Mo	0.65 (0.24, 1.29)	0.67 (0.31, 1.37)	0.58 (0.31, 1.13)	0.76 (0.44, 1.25)	1.53 (0.95, 2.75)
1.54 (0.77, 4.24)	DAPT 1Mo+P2Y12	1.03 (0.37, 3.63)	0.89 (0.36, 3.08)	1.18 (0.49, 3.53)	**2.38 (1.05, 8.20)**
1.50 (0.73, 3.26)	0.97 (0.28, 2.67)	DAPT 3Mo+ASA	0.86 (0.34, 2.41)	1.13 (0.46, 2.90)	**2.30 (1.00, 6.28)**
1.73 (0.89, 3.27)	1.12 (0.32, 2.78)	1.16 (0.41, 2.93)	DAPT 3Mo+P2Y12	1.31 (0.54, 2.88)	**2.66 (1.17, 6.36)**
1.32 (0.80, 2.27)	0.85 (0.28, 2.02)	0.89 (0.35, 2.18)	0.76 (0.35, 1.85)	DAPT 6Mo	**2.04 (1.17, 3.93)**
0.65 (0.36, 1.05)	**0.42 (0.12, 0.95)**	**0.44 (0.16, 1.00)**	**0.38 (0.16, 0.85)**	**0.49 (0.25, 0.85)**	L-DAPT
**Any bleeding**					
DAPT 12Mo	0.75 (0.37, 1.16)	0.73 (0.43, 1.21)	**0.58 (0.37, 0.87)**	**0.72 (0.50, 1.00)**	**1.53 (1.05, 2.12)**
1.33 (0.87, 2.72)	DAPT 1Mo+P2Y12	0.98 (0.50, 2.40)	0.76 (0.43, 1.79)	0.97 (0.55, 2.10)	**2.02 (1.18, 4.33)**
1.37 (0.82, 2.31)	1.02 (0.42, 1.99)	DAPT 3Mo+ASA	0.79 (0.41, 1.52)	0.99 (0.53, 1.84)	**2.10 (1.09, 3.84)**
**1.74 (1.15, 2.70)**	1.32 (0.56, 2.33)	1.27 (0.66, 2.45)	DAPT 3Mo+P2Y12	1.26 (0.72, 2.16)	**2.67 (1.50, 4.52)**
**1.39 (1.00, 1.99)**	1.04 (0.48, 1.80)	1.01 (0.54, 1.90)	0.79 (0.46, 1.39)	DAPT 6Mo	**2.10 (1.42, 3.10)**
**0.65 (0.47, 0.96)**	**0.50 (0.23, 0.85)**	**0.48 (0.26, 0.91)**	**0.37 (0.22, 0.67)**	**0.48 (0.32, 0.71)**	L-DAPT
**Definite or probable stent thrombosis**					
DAPT 12Mo	1.23 (0.52, 3.79)	1.29 (0.52, 3.06)	1.00 (0.43, 2.64)	1.15 (0.62, 2.28)	0.56 (0.32, 1.11)
0.81 (0.26, 1.93)	DAPT 1Mo+P2Y12	1.04 (0.24, 3.40)	0.80 (0.20, 2.94)	0.91 (0.27, 2.86)	0.45 (0.14, 1.38)
0.78 (0.33, 1.91)	0.96 (0.29, 4.21)	DAPT 3Mo+ASA	0.79 (0.24, 2.99)	0.90 (0.32, 2.81)	0.45 (0.16, 1.37)
1.00 (0.38, 2.34)	1.26 (0.34, 4.92)	1.27 (0.33, 4.13)	DAPT 3Mo+P2Y12	1.12 (0.37, 3.34)	0.57 (0.19, 1.67)
0.87 (0.44, 1.61)	1.10 (0.35, 3.76)	1.12 (0.36, 3.15)	0.90 (0.30, 2.67)	DAPT 6Mo	0.50 (0.25, 1.01)
1.77 (0.90, 3.10)	2.21 (0.72, 7.35)	2.23 (0.73, 6.21)	1.77 (0.60, 5.24)	2.00 (0.99, 4.02)	L-DAPT
**Net adverse clinical events**					
DAPT 12Mo	0.88 (0.66, 1.07)	1.00 (0.78, 1.25)	**0.71 (0.54, 0.96)**	1.04 (0.85, 1.26)	0.98 (0.77, 1.22)
1.14 (0.93, 1.51)	DAPT 1Mo+P2Y12	1.14 (0.83, 1.64)	0.81 (0.58, 1.25)	1.19 (0.88, 1.66)	1.12 (0.81, 1.56)
1.00 (0.80, 1.28)	0.88 (0.61, 1.21)	DAPT 3Mo+ASA	0.72 (0.50, 1.06)	1.05 (0.76, 1.42)	0.98 (0.71, 1.36)
**1.40 (1.04, 1.85)**	1.24 (0.80, 1.72)	1.39 (0.94, 1.98)	DAPT 3Mo+P2Y12	**1.46 (1.02, 2.02)**	1.37 (0.93, 1.93)

0.96 (0.79, 1.18)	0.84 (0.60, 1.13)	0.95 (0.71, 1.32)	**0.69 (0.50, 0.98)**	DAPT 6Mo	0.93 (0.73, 1.22)
1.02 (0.82, 1.31)	0.89 (0.64, 1.23)	1.02 (0.74, 1.40)	0.73 (0.52, 1.07)	1.07 (0.82, 1.38)	L-DAPT

Supplementary Table S11 The number of participants with acute coronary syndrome who had each outcome

Trial	Treatment	Total	All-cause mortality	MI	Major bleeding	Any bleeding	Definite or probable ST	NACE
TICO	DAPT 3Mo+P2Y12	1527	16	6	25	53	6	59
	DAPT 12Mo	1529	23	11	45	83	4	89
GLOBAL LEADERS	DAPT 1Mo+P2Y12	3750	38	50	28	97	7	248
	DAPT 12Mo	3737	51	52	54	152	6	266
REDUCE	DAPT 3Mo+ASA	733	23	26	24	24	12	85
	DAPT 12Mo	727	16	22	29	29	6	88
DAPT-STEMI	DAPT 6Mo	433	3	8	1	3	3	21
	DAPT 12Mo	437	6	8	2	5	4	29
IVUS-XPL+EXCELLENT+RESET	DAPT 6Mo	1119	9	7	4	9	6	22
	DAPT 12Mo	1097	11	5	6	14	4	21
SMART-DATE	DAPT 6Mo	1357	35	24	6	35	15	96
	L-DAPT	1355	39	10	10	51	10	99
ITALIC	DAPT 6Mo	400	4	7	0	2	5	14
	L-DAPT	406	9	6	2	6	3	17
PRODIGY	L-DAPT	733	56	39	17	30	15	103
	DAPT 6Mo	732	52	33	23	52	10	118
ISAR-SAFE	DAPT 6Mo	794	5	6	1	3	2	14
	DAPT 12Mo	807	7	8	2	5	2	17

DAPT Study	DAPT 12Mo	1771	27	88	14	35	32	NA
	L-DAPT	1805	24	39	34	76	9	NA
TWILIGHT	DAPT 3Mo+P2Y12	2273	22	70	17	81	8	NA
	DAPT 12Mo	2341	34	72	49	175	14	NA

NA, the concrete outcomes was not available; NACE, net adverse clinical events; MI, myocardial infarction; ST, stent thrombosis; DAPT, dual antiplatelet therapy; L-DAPT, longer than 12 months DAPT; DAPT 12Mo,12 months DAPT; DAPT 6Mo,6 months DAPT; DAPT 3Mo+ASA, 3-months DAPT followed by aspirin monotherapy; DAPT 3Mo+P2Y12, 3-month DAPT followed by a P2Y12 receptor inhibitor monotherapy; DAPT 1Mo+P2Y12,1-month DAPT followed by a P2Y12 receptor inhibitor monotherapy.

Supplementary Table S12 Pooled estimates of the network meta-analysis of each endpoint in participants with acute coronary syndrome

All-cause mortality

DAPT 12Mo	0.74 (0.39, 1.43)	1.44 (0.63, 3.45)	0.68 (0.39, 1.17)	0.75 (0.43, 1.25)	0.83 (0.48, 1.45)
1.36 (0.70, 2.59)	DAPT 1Mo+P2Y12	1.96 (0.68, 5.58)	0.92 (0.39, 2.16)	1.02 (0.42, 2.33)	1.14 (0.47, 2.63)
0.69 (0.29, 1.59)	0.51 (0.18, 1.47)	DAPT 3Mo+ASA	0.46 (0.17, 1.23)	0.52 (0.18, 1.35)	0.57 (0.21, 1.56)
1.47 (0.85, 2.55)	1.09 (0.46, 2.59)	2.16 (0.81, 6.02)	DAPT 3Mo+P2Y12	1.12 (0.50, 2.32)	1.23 (0.56, 2.68)
1.33 (0.80, 2.34)	0.98 (0.43, 2.41)	1.92 (0.74, 5.41)	0.89 (0.43, 2.02)	DAPT 6Mo	1.10 (0.77, 1.73)
1.20 (0.69, 2.09)	0.88 (0.38, 2.11)	1.74 (0.64, 4.75)	0.81 (0.37, 1.77)	0.91 (0.58, 1.30)	L-DAPT

Myocardial infarction

DAPT 12Mo	0.96 (0.41, 2.23)	1.16 (0.45, 2.98)	0.86 (0.40, 1.62)	0.82 (0.46, 1.59)	**0.49 (0.27, 0.98)**
1.04 (0.45, 2.47)	DAPT 1Mo+P2Y12	1.23 (0.35, 4.46)	0.90 (0.27, 2.59)	0.84 (0.32, 2.58)	0.51 (0.19, 1.55)
0.86 (0.34, 2.21)	0.81 (0.22, 2.85)	DAPT 3Mo+ASA	0.74 (0.21, 2.36)	0.70 (0.24, 2.23)	0.42 (0.14, 1.37)
1.16 (0.62, 2.50)	1.11 (0.39, 3.64)	1.35 (0.42, 4.74)	DAPT 3Mo+P2Y12	0.95 (0.42, 2.75)	0.57 (0.24, 1.65)
1.22 (0.63, 2.16)	1.19 (0.39, 3.13)	1.43 (0.45, 4.21)	1.05 (0.36, 2.41)	DAPT 6Mo	0.61 (0.35, 1.03)
**2.03 (1.02, 3.66)**	1.95 (0.65, 5.38)	2.37 (0.73, 7.09)	1.75 (0.61, 4.09)	1.65 (0.97, 2.87)	L-DAPT

Major bleeding

DAPT 12Mo	0.51 (0.14, 1.71)	0.81 (0.22, 2.88)	0.44 (0.18, 1.05)	0.91 (0.29, 2.05)	1.83 (0.65, 4.37)
1.95 (0.59, 6.90)	DAPT 1Mo+P2Y12	1.59 (0.27, 9.33)	0.87 (0.19, 3.92)	1.80 (0.32, 6.92)	3.61 (0.74, 16.18)
1.23 (0.35, 4.51)	0.63 (0.11, 3.77)	DAPT 3Mo+ASA	0.54 (0.12, 2.57)	1.14 (0.18, 4.52)	2.21 (0.41, 10.06)
2.27 (0.96, 5.48)	1.15 (0.26, 5.20)	1.85 (0.39, 8.56)	DAPT 3Mo+P2Y12	2.06 (0.48, 6.29)	**4.20 (1.08, 13.91)**
1.10 (0.49, 3.43)	0.56 (0.14, 3.17)	0.88 (0.22, 5.43)	0.49 (0.16, 2.07)	DAPT 6Mo	2.01 (0.97, 5.15)

0.55 (0.23, 1.54)	0.28 (0.06, 1.35)	0.45 (0.10, 2.41)	**0.24 (0.07, 0.93)**	0.50 (0.19, 1.03)	L-DAPT

Any bleeding

DAPT 12Mo	0.63 (0.30, 1.36)	0.81 (0.33, 1.92)	**0.52 (0.31, 0.92)**	0.87 (0.43, 1.51)	1.72 (0.89, 3.02)
1.58 (0.74, 3.37)	DAPT 1Mo+P2Y12	1.28 (0.40, 4.03)	0.83 (0.33, 2.16)	1.41 (0.46, 3.33)	2.72 (0.94, 6.80)
1.23 (0.52, 3.01)	0.78 (0.25, 2.50)	DAPT 3Mo+ASA	0.65 (0.24, 1.85)	1.09 (0.34, 2.95)	2.12 (0.68, 5.95)
**1.91 (1.09, 3.19)**	1.20 (0.46, 2.99)	1.54 (0.54, 4.17)	DAPT 3Mo+P2Y12	1.68 (0.65, 3.42)	**3.28 (1.34, 6.88)**
1.15 (0.66, 2.32)	0.71 (0.30, 2.16)	0.92 (0.34, 2.98)	0.60 (0.29, 1.54)	DAPT 6Mo	**1.95 (1.25, 3.37)**
0.58 (0.33, 1.12)	0.37 (0.15, 1.07)	0.47 (0.17, 1.47)	**0.31 (0.15, 0.75)**	**0.51 (0.30, 0.80)**	L-DAPT

Definite or probable stent thrombosis

DAPT 12Mo	1.18 (0.28, 5.45)	2.06 (0.51, 8.65)	0.80 (0.31, 2.27)	0.73 (0.33, 1.71)	**0.40 (0.16, 0.95)**
0.85 (0.18, 3.63)	DAPT 1Mo+P2Y12	1.67 (0.23, 12.83)	0.66 (0.11, 4.32)	0.61 (0.10, 3.30)	0.34 (0.06, 1.84)
0.49 (0.12, 1.96)	0.60 (0.08, 4.38)	DAPT 3Mo+ASA	0.40 (0.07, 2.22)	0.35 (0.07, 1.91)	0.19 (0.04, 1.03)
1.24 (0.44, 3.18)	1.52 (0.23, 8.72)	2.51 (0.45, 13.71)	DAPT 3Mo+P2Y12	0.90 (0.26, 3.20)	0.49 (0.13, 1.75)
1.38 (0.59, 3.00)	1.63 (0.30, 9.63)	2.85 (0.52, 14.22)	1.11 (0.31, 3.88)	DAPT 6Mo	0.54 (0.26, 1.05)
**2.51 (1.05, 6.12)**	2.95 (0.54, 17.88)	5.21 (0.97, 26.99)	2.04 (0.57, 7.72)	1.84 (0.96, 3.80)	L-DAPT

Net adverse clinical events

DAPT 12Mo	0.92 (0.61, 1.41)	0.95 (0.58, 1.54)	0.65 (0.40, 1.07)	0.83 (0.57, 1.28)	0.93 (0.57, 1.61)
1.09 (0.71, 1.64)	DAPT 1Mo+P2Y12	1.04 (0.54, 1.96)	0.70 (0.37, 1.33)	0.92 (0.52, 1.62)	1.01 (0.54, 1.98)
1.05 (0.65, 1.72)	0.96 (0.51, 1.84)	DAPT 3Mo+ASA	0.67 (0.34, 1.35)	0.88 (0.48, 1.65)	0.98 (0.50, 2.00)
1.54 (0.94, 2.48)	1.42 (0.75, 2.69)	1.48 (0.74, 2.92)	DAPT 3Mo+P2Y12	1.30 (0.71, 2.42)	1.45 (0.73, 2.90)
1.20 (0.78, 1.76)	1.09 (0.62, 1.91)	1.14 (0.60, 2.09)	0.77 (0.41, 1.41)	DAPT 6Mo	1.11 (0.83, 1.51)
1.07 (0.62, 1.76)	0.99 (0.51, 1.85)	1.02 (0.50, 1.99)	0.69 (0.34, 1.37)	0.90 (0.66, 1.20)	L-DAPT

Supplementary Table S13 The number of participants with newer-generation drug-eluting stents who had each outcome

Trial	Treatment	Total	All-cause mortality	MI	Major bleeding	Any bleeding	Definite or probable ST	NACE
TICO	DAPT 3Mo+P2Y12	1527	16	6	25	53	6	59
	DAPT 12Mo	1529	23	11	45	83	4	89
STOPDAPT-2	DAPT 1Mo+P2Y12	1500	21	13	3	6	4	35
	DAPT 12Mo	1509	18	11	16	23	1	55
TWILIGHT	DAPT 3Mo+P2Y12	3555	34	95	34	141	14	NA
	DAPT 12Mo	3564	45	95	69	250	19	NA
SMART-CHOICE	DAPT 3Mo+P2Y12	1495	21	11	12	28	3	65
	DAPT 12Mo	1498	18	17	14	49	2	81
REDUCE	DAPT 3Mo+ASA	733	23	26	24	24	12	85
	DAPT 12Mo	727	16	22	29	29	6	88
GLOBAL LEADERS	DAPT 1Mo+P2Y12	7980	224	248	163	529	64	616
	DAPT 12Mo	7988	253	250	169	532	64	653
OPTIMA-C	DAPT 6Mo	683	2	1	1	1	0	NA
	DAPT 12Mo	684	3	1	1	1	1	NA
DAPT-STEMI	DAPT 6Mo	433	3	8	1	3	3	21
	DAPT 12Mo	437	6	8	2	5	4	29

SMART-DATE	DAPT 6Mo	1357	35	24	6	35	15	96
	L-DAPT	1355	39	10	10	51	10	99
NIPPON	L-DAPT	1653	7	1	12	12	1	24
	DAPT 6Mo	1654	16	4	11	11	2	34
IVUS-XPL	DAPT 6Mo	699	5	1	5	5	2	15
	DAPT 12Mo	701	10	0	7	7	2	14
I-LOVE-IT 2	DAPT 6Mo	909	14	42	13	57	5	72
	DAPT 12Mo	920	17	37	7	60	2	67
ITALIC	DAPT 6Mo	926	11	12	0	6	6	32
	L-DAPT	924	20	9	4	10	3	34
DAPT Study	L-DAPT	2345	49	48	21	57	6	NA
	DAPT 12Mo	2358	26	72	7	30	16	NA
SECURITY	DAPT 6Mo	682	14	21	5	6	3	46
	DAPT 12Mo	717	15	19	8	10	3	45
OPTIMIZE	DAPT 3Mo+ASA	1563	43	49	10	35	13	93
	DAPT 12Mo	1556	45	42	14	45	12	90
PRODIGY	L-DAPT	496	30	16	NA	NA	4	NA
	DAPT 6Mo	492	25	12	NA	NA	1	NA
EXCELLENT	DAPT 6Mo	540	3	9	2	3	3	16
	DAPT 12Mo	539	4	6	3	9	1	16

NA, the concrete outcomes was not available; MI, myocardial infarction; ST, stent thrombosis; ANCE, net adverse clinical events; DAPT, dual antiplatelet therapy; L-DAPT, longer than 12 months DAPT; DAPT 12Mo,12 months DAPT; DAPT 6Mo,6 months DAPT; DAPT 3Mo+ASA, 3-months DAPT followed by aspirin monotherapy; DAPT 3Mo+P2Y12, 3-month DAPT followed by a P2Y12 receptor inhibitor monotherapy; DAPT 1Mo+P2Y12,1-month DAPT followed by a P2Y12 receptor inhibitor monotherapy.

Supplementary Table S14 Pooled estimates of the network meta-analysis of each endpoint in Participants with newer-generation drug-eluting stents

All-cause mortality

DAPT 12Mo	0.94 (0.59, 1.64)	1.10 (0.64, 1.96)	0.83 (0.52, 1.34)	0.94 (0.57, 1.45)	1.20 (0.68, 1.88)
1.07 (0.61, 1.71)	DAPT 1Mo+P2Y12	1.17 (0.53, 2.44)	0.87 (0.43, 1.73)	0.99 (0.47, 1.80)	1.27 (0.55, 2.35)
0.91 (0.51, 1.57)	0.86 (0.41, 1.88)	DAPT 3Mo+ASA	0.74 (0.36, 1.56)	0.86 (0.39, 1.63)	1.09 (0.47, 2.13)
1.21 (0.75, 1.93)	1.15 (0.58, 2.34)	1.34 (0.64, 2.76)	DAPT 3Mo+P2Y12	1.13 (0.56, 2.07)	1.45 (0.68, 2.73)
1.06 (0.69, 1.74)	1.01 (0.56, 2.15)	1.16 (0.61, 2.59)	0.88 (0.48, 1.78)	DAPT 6Mo	1.27 (0.86, 1.87)
0.83 (0.53, 1.47)	0.79 (0.43, 1.81)	0.91 (0.47, 2.14)	0.69 (0.37, 1.47)	0.79 (0.53, 1.16)	L-DAPT

Myocardial infarction

DAPT 12Mo	1.01 (0.70, 1.55)	1.16 (0.75, 1.83)	0.87 (0.55, 1.23)	1.14 (0.80, 1.64)	0.71 (0.48, 1.08)
0.99 (0.65, 1.43)	DAPT 1Mo+P2Y12	1.15 (0.62, 2.07)	0.86 (0.44, 1.40)	1.13 (0.66, 1.87)	0.70 (0.40, 1.23)
0.86 (0.55, 1.34)	0.87 (0.48, 1.60)	DAPT 3Mo+ASA	0.74 (0.39, 1.30)	0.98 (0.54, 1.74)	0.62 (0.33, 1.13)
1.15 (0.82, 1.81)	1.16 (0.72, 2.25)	1.35 (0.77, 2.58)	DAPT 3Mo+P2Y12	1.30 (0.82, 2.38)	0.83 (0.50, 1.57)
0.88 (0.61, 1.25)	0.89 (0.53, 1.51)	1.02 (0.57, 1.86)	0.77 (0.42, 1.22)	DAPT 6Mo	**0.63 (0.42, 0.93)**
1.40 (0.92, 2.07)	1.42 (0.82, 2.53)	1.61 (0.89, 3.00)	1.21 (0.64, 2.01)	**1.59 (1.07, 2.37)**	L-DAPT

Major bleeding

DAPT 12Mo	0.65 (0.21, 1.31)	0.78 (0.31, 1.84)	0.58 (0.29, 1.21)	1.05 (0.50, 1.98)	2.13 (0.93, 5.37)
1.54 (0.76, 4.69)	DAPT 1Mo+P2Y12	1.22 (0.41, 5.27)	0.89 (0.35, 3.59)	1.63 (0.59, 5.61)	**3.34 (1.15, 14.66)**
1.28 (0.54, 3.23)	0.82 (0.19, 2.47)	DAPT 3Mo+ASA	0.74 (0.25, 2.47)	1.34 (0.43, 4.09)	2.75 (0.83, 10.16)
1.73 (0.83, 3.39)	1.12 (0.28, 2.85)	1.35 (0.40, 4.04)	DAPT 3Mo+P2Y12	1.82 (0.62, 4.48)	**3.69 (1.22, 11.42)**
0.95 (0.50, 1.98)	0.61 (0.18, 1.68)	0.74 (0.24, 2.31)	0.55 (0.22, 1.60)	DAPT 6Mo	**2.04 (1.01, 5.02)**

0.47 (0.19, 1.07)	**0.30 (0.07, 0.87)**	0.36 (0.10, 1.20)	**0.27 (0.09, 0.82)**	**0.49 (0.20, 0.99)**	L-DAPT

Any bleeding

DAPT 12Mo	0.79 (0.37, 1.17)	0.79 (0.45, 1.40)	**0.57 (0.38, 0.88)**	0.85 (0.53, 1.25)	1.42 (0.79, 2.30)
1.26 (0.86, 2.74)	DAPT 1Mo+P2Y12	1.00 (0.52, 2.66)	0.72 (0.43, 1.83)	1.09 (0.61, 2.43)	1.81 (0.95, 4.31)
1.27 (0.72, 2.24)	1.00 (0.38, 1.92)	DAPT 3Mo+ASA	0.73 (0.36, 1.48)	1.08 (0.49, 2.08)	1.82 (0.78, 3.75)
**1.75 (1.14, 2.64)**	1.39 (0.55, 2.30)	1.38 (0.68, 2.79)	DAPT 3Mo+P2Y12	1.49 (0.78, 2.56)	**2.49 (1.20, 4.57)**
1.18 (0.80, 1.88)	0.92 (0.41, 1.63)	0.93 (0.48, 2.02)	0.67 (0.39, 1.29)	DAPT 6Mo	**1.67 (1.05, 2.68)**
0.70 (0.43, 1.27)	0.55 (0.23, 1.05)	0.55 (0.27, 1.28)	**0.40 (0.22, 0.83)**	**0.60 (0.37, 0.95)**	L-DAPT

Definite or probable stent thrombosis

DAPT 12Mo	1.16 (0.57, 3.30)	1.38 (0.60, 3.51)	0.98 (0.46, 2.33)	0.91 (0.47, 2.01)	0.57 (0.25, 1.45)
0.86 (0.30, 1.76)	DAPT 1Mo+P2Y12	1.17 (0.30, 3.60)	0.83 (0.23, 2.57)	0.78 (0.23, 2.28)	0.49 (0.13, 1.56)
0.72 (0.29, 1.68)	0.85 (0.28, 3.39)	DAPT 3Mo+ASA	0.71 (0.22, 2.42)	0.69 (0.21, 2.18)	0.41 (0.13, 1.52)
1.02 (0.43, 2.18)	1.20 (0.39, 4.37)	1.40 (0.41, 4.58)	DAPT 3Mo+P2Y12	0.93 (0.33, 2.74)	0.58 (0.19, 1.95)
1.09 (0.50, 2.14)	1.28 (0.44, 4.41)	1.46 (0.46, 4.72)	1.08 (0.36, 3.03)	DAPT 6Mo	0.62 (0.31, 1.28)
1.74 (0.69, 3.93)	2.06 (0.64, 7.74)	2.42 (0.66, 7.88)	1.72 (0.51, 5.38)	1.62 (0.78, 3.24)	L-DAPT

Net adverse clinical events

DAPT 12Mo	0.88 (0.62, 1.10)	1.00 (0.72, 1.34)	**0.72 (0.52, 0.99)**	1.00 (0.77, 1.29)	0.95 (0.63, 1.39)
1.14 (0.91, 1.61)	DAPT 1Mo+P2Y12	1.14 (0.77, 1.80)	0.82 (0.57, 1.32)	1.15 (0.81, 1.74)	1.09 (0.69, 1.79)
1.00 (0.74, 1.39)	0.88 (0.56, 1.30)	DAPT 3Mo+ASA	0.72 (0.47, 1.14)	1.00 (0.67, 1.53)	0.95 (0.58, 1.60)
**1.40 (1.01, 1.91)**	1.22 (0.76, 1.76)	1.38 (0.88, 2.14)	DAPT 3Mo+P2Y12	1.39 (0.92, 2.06)	1.32 (0.80, 2.18)
1.00 (0.78, 1.30)	0.87 (0.58, 1.24)	1.00 (0.65, 1.49)	0.72 (0.49, 1.08)	DAPT 6Mo	0.96 (0.70, 1.26)
1.05 (0.72, 1.58)	0.92 (0.56, 1.45)	1.05 (0.63, 1.73)	0.76 (0.46, 1.26)	1.05 (0.79, 1.43)	L-DAPT

Supplementary Fig. S1 Pair-wise meta-analysis of all endpoints

All-cause mortality

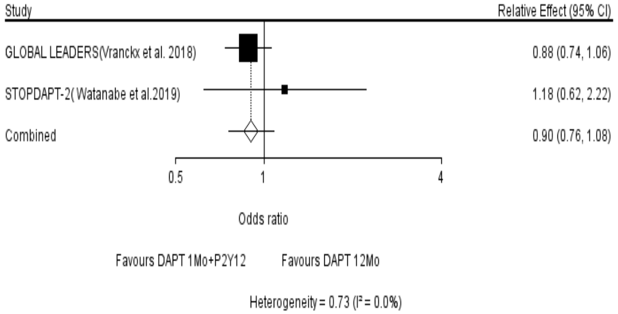

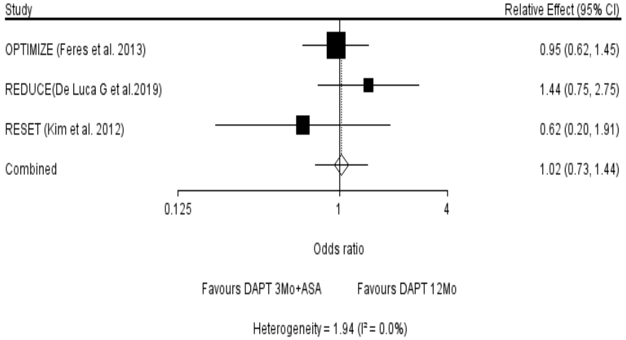

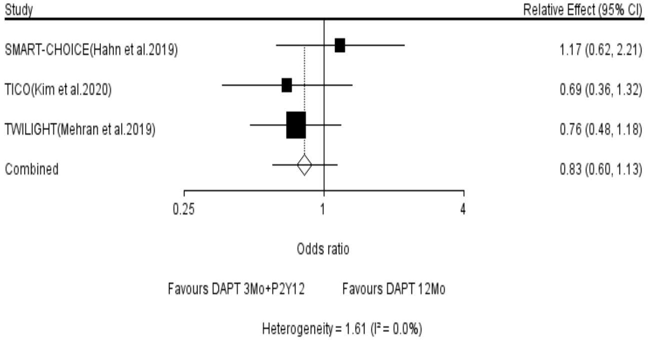

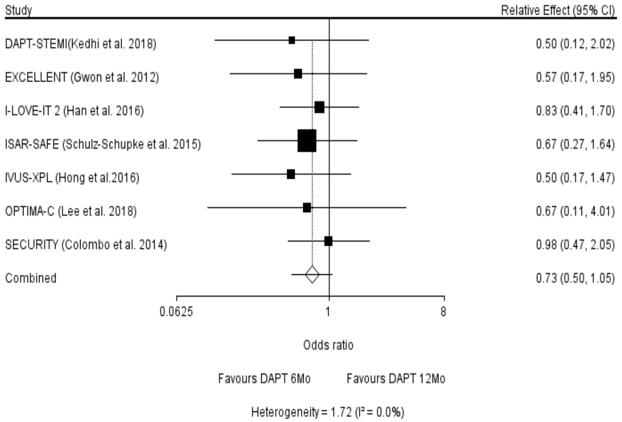

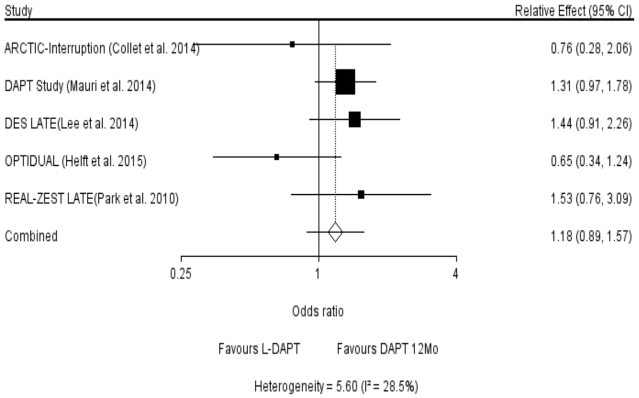

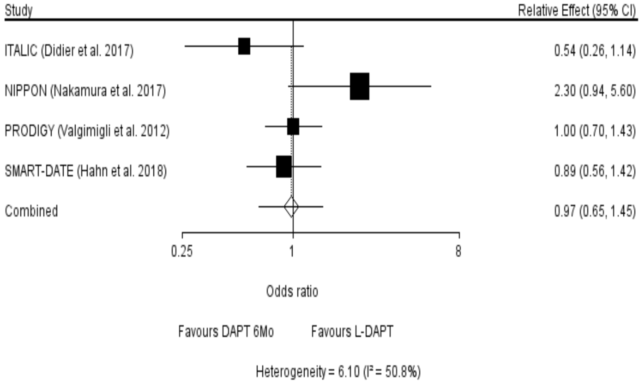

Cardiac death

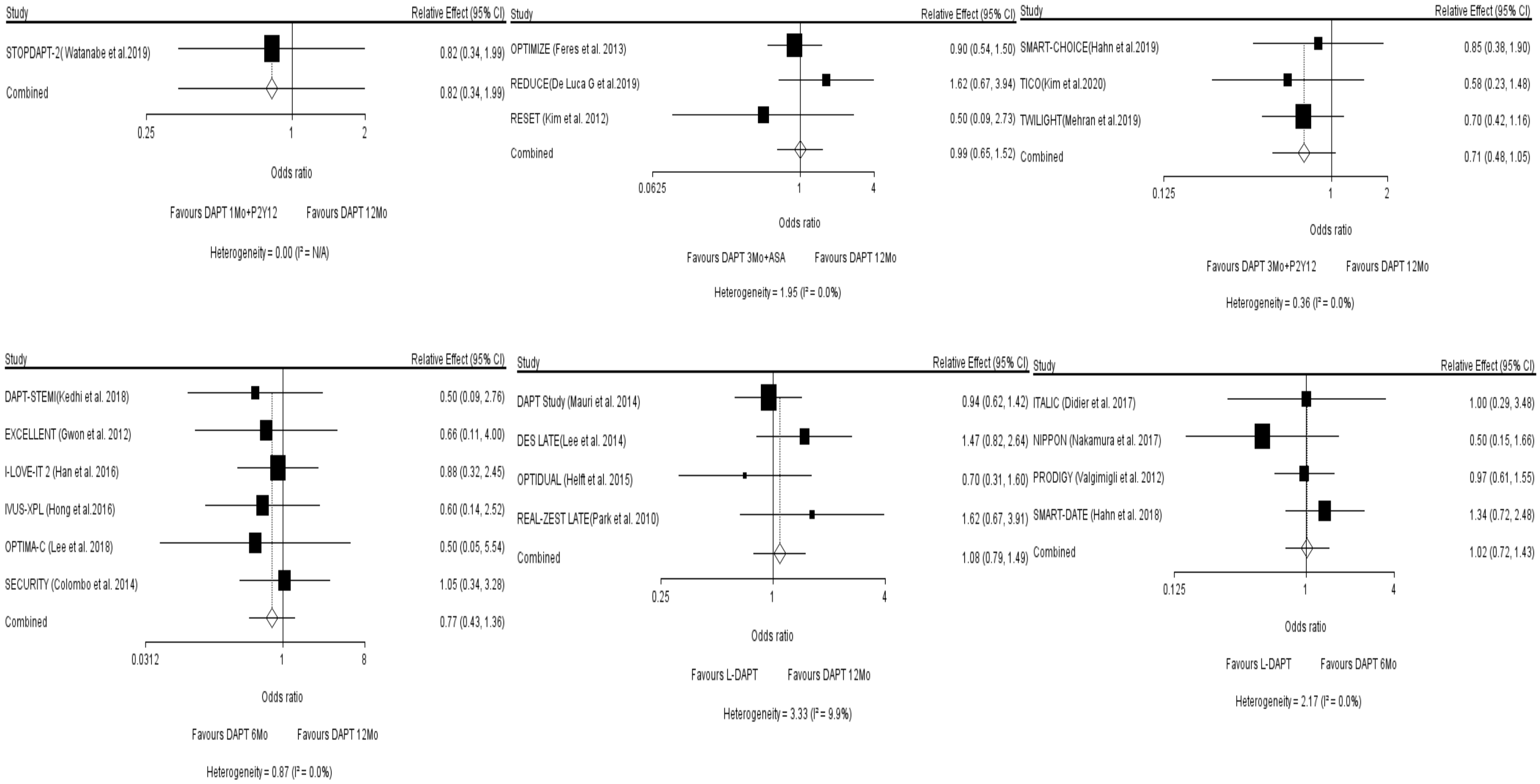

Myocardial infarction

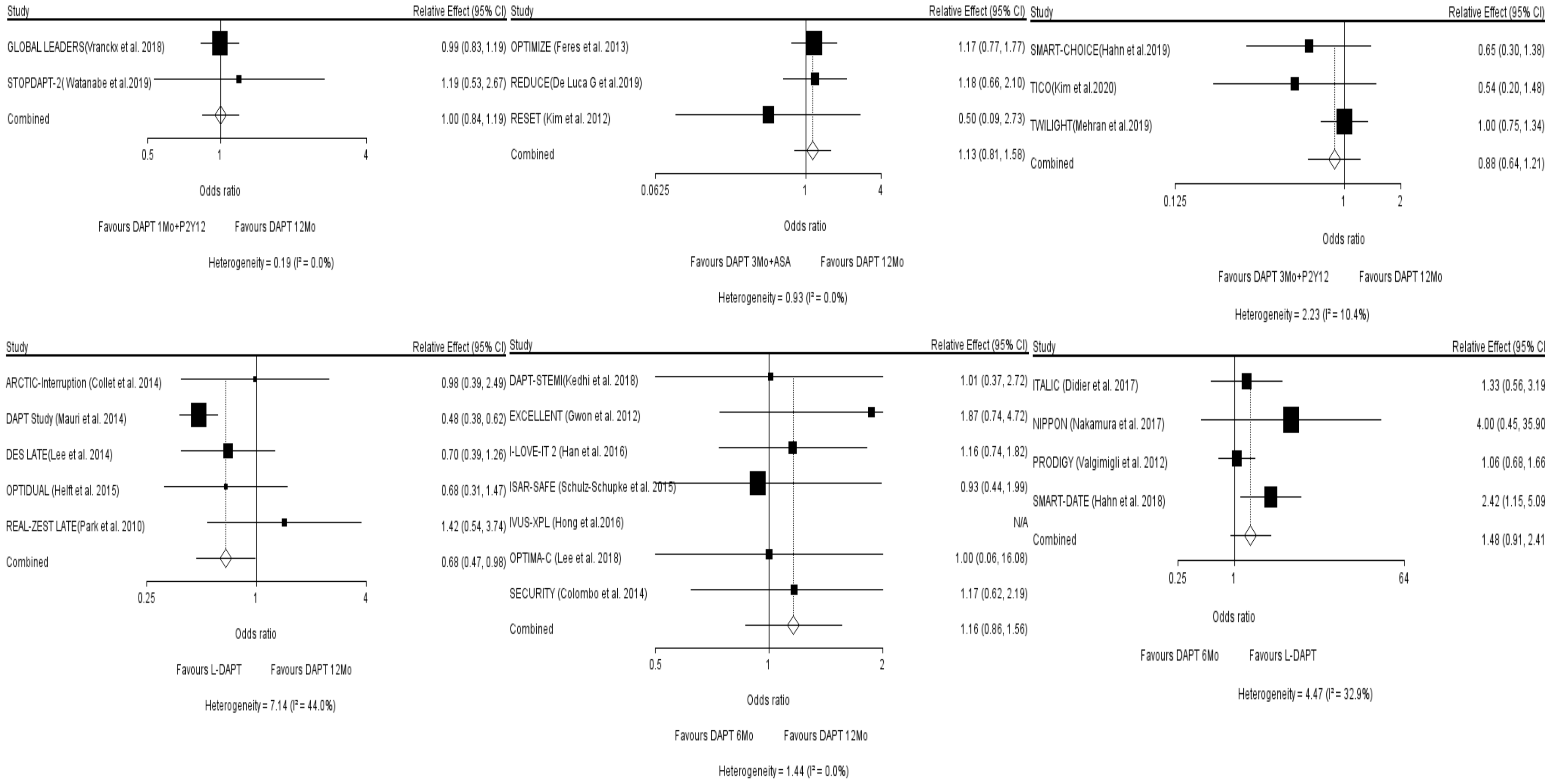

Major bleeding

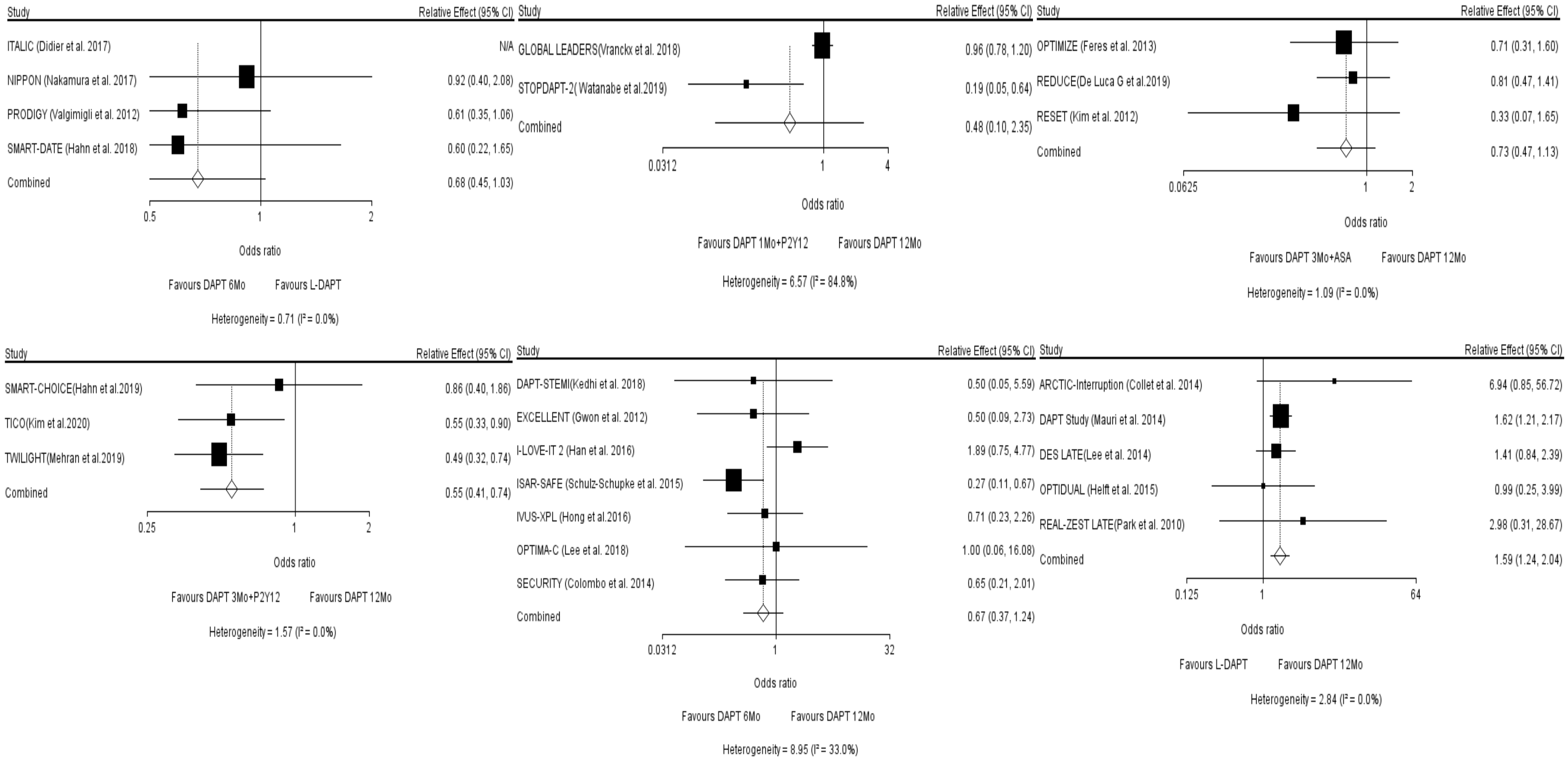

Any bleeding

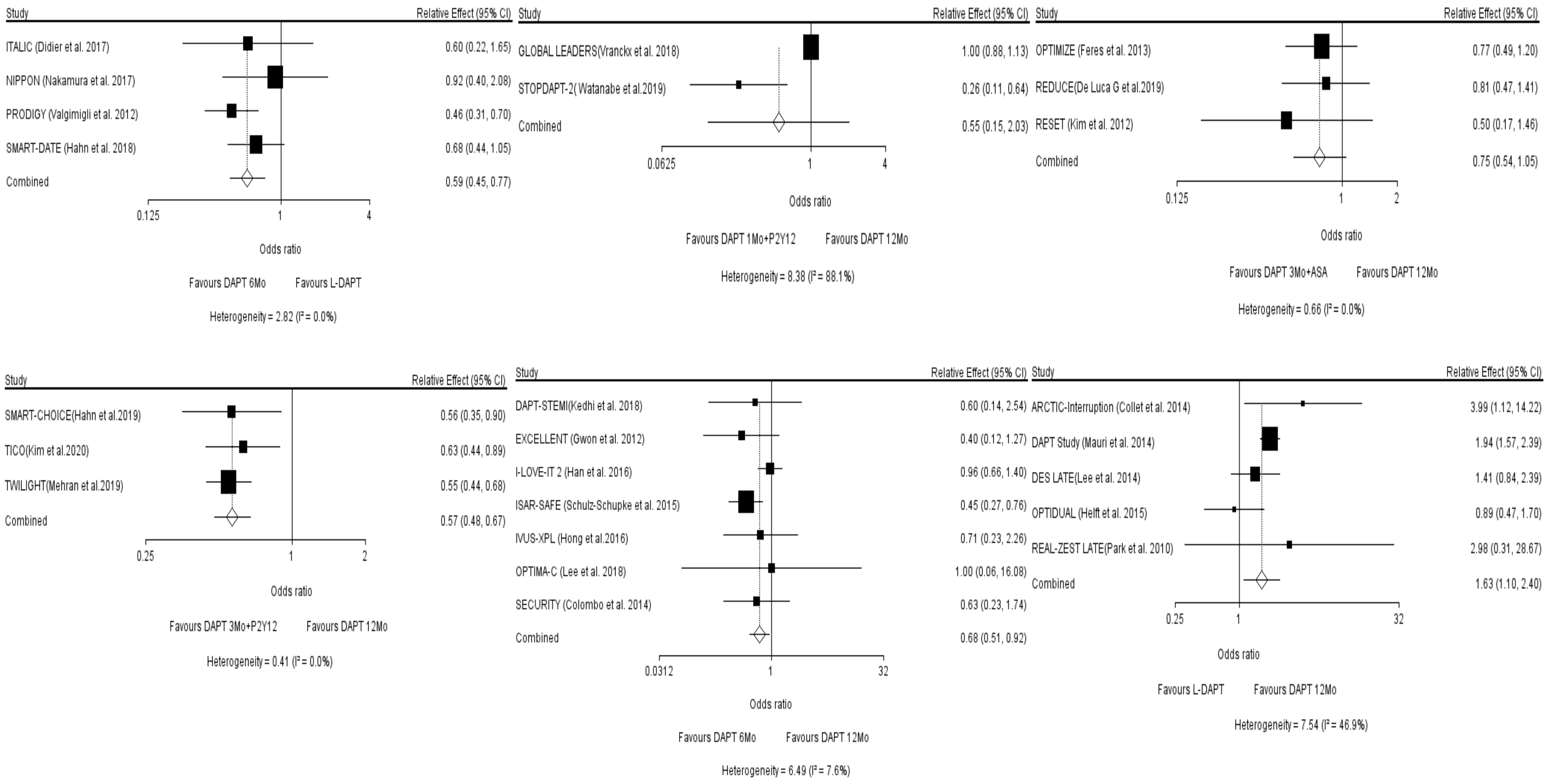

Definite or probable stent thrombosis

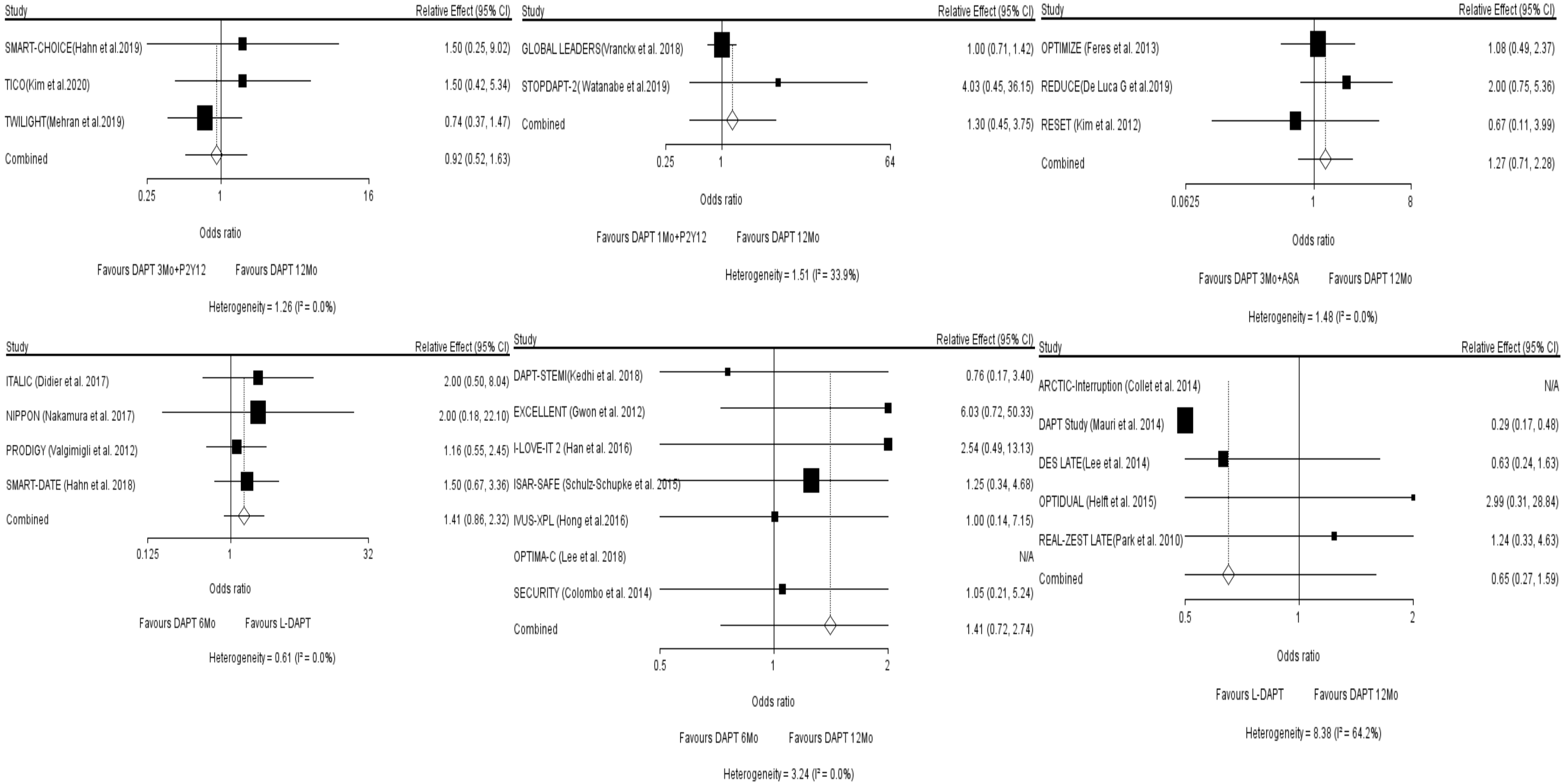

Net adverse clinical events

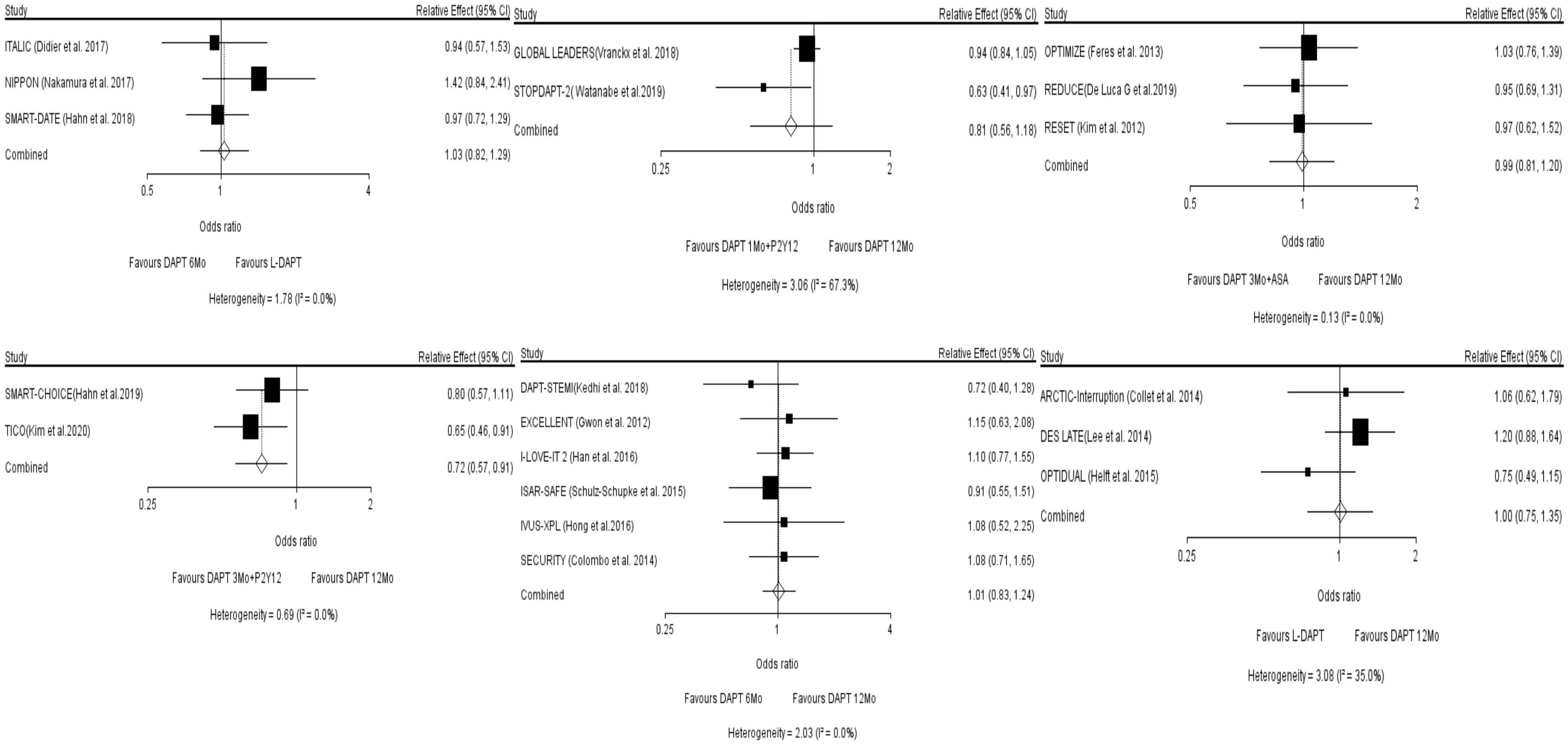

Supplementary Fig. S2 Funnel plots of each endpoint. A refers to DAPT 12Mo, B refers to DAPT 1Mo+P2Y12, C refers to DAPT 3Mo+ASA, D refers to DAPT 3Mo+P2Y12, E refers to DAPT 6Mo, and F refers to L-DAPT.

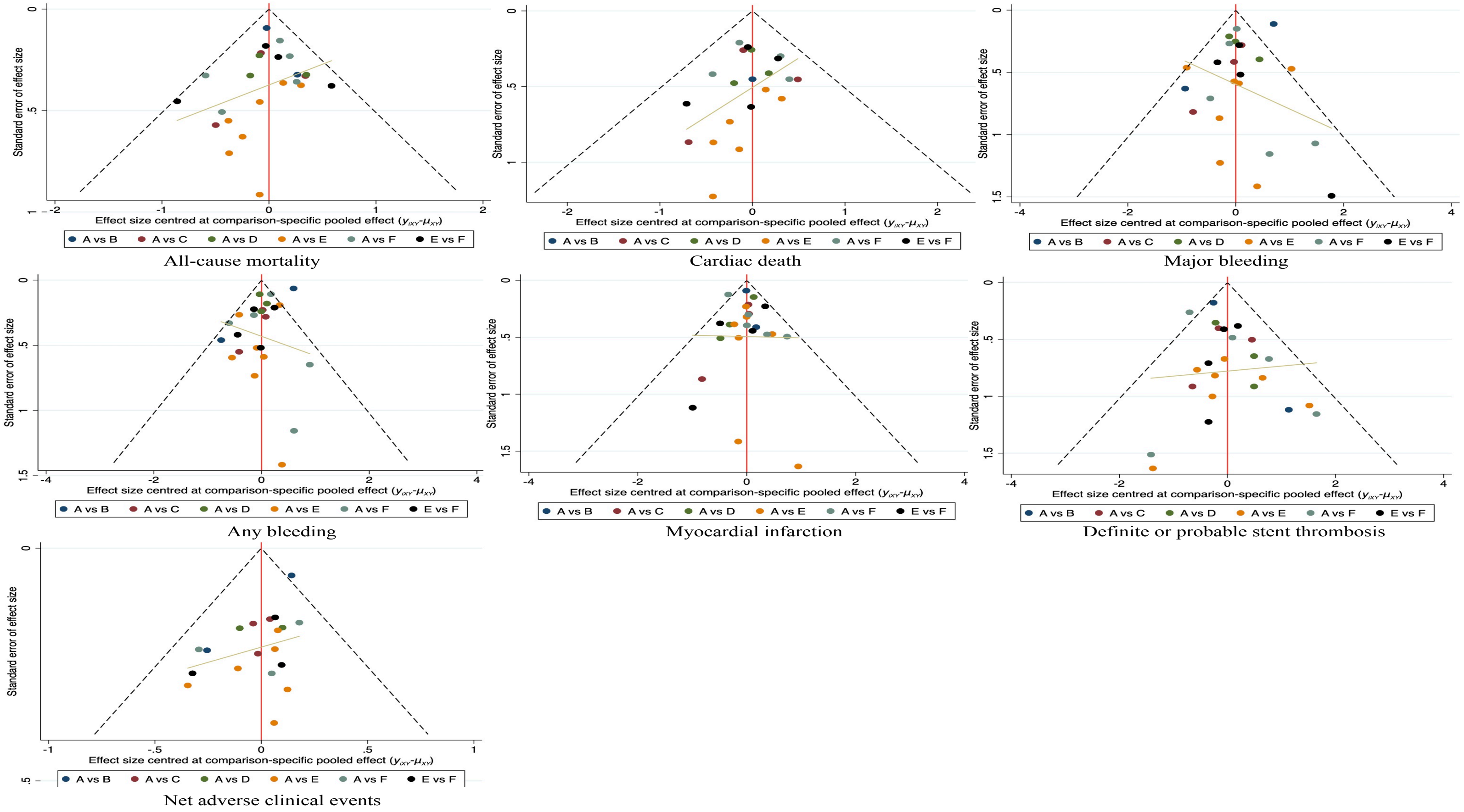

Supplement: Supplementary Materials — Supplementary Table S1. PRISMA checklist; Supplementary Table S2. Individual search algorithm; Supplementary Table S3. Baseline characteristics of the included trials; Supplementary Table S4. Baseline characteristics of participants; Supplementary Table S5. Definitions of clinical endpoints; Supplementary Table S6. The number of participants who had each outcome; Supplementary Table S7. Risk of bias of included trials; Supplementary Table S8. Pooled estimates of the network meta-analysis; Supplementary Table S9. Node-splitting analysis of inconsistency; Supplementary Table S10. Pooled estimates of sensitivity analysis; Supplementary Table S11. The number of participants with acute coronary syndrome; Supplementary Table S12. Pooled estimates of the network meta-analysis with acute coronary syndrome; Supplementary Table S13. The number of participants with newer-generation drug-eluting stents; Supplementary Table S14. Pooled estimates of the network meta-analysis with newer-generation drug-eluting stents; Supplementary Figure S1. Pair-wise meta-analysis of all endpoints; Supplementary Figure S2. Funnel plots. [file 9934535.f1.pdf]
